# Molecular Modeling and In Vitro Evaluation of Piplartine Analogs against Oral Squamous Cell Carcinoma

**DOI:** 10.3390/molecules28041675

**Published:** 2023-02-09

**Authors:** Rayanne H. N. Silva, Thaíssa Q. Machado, Anna Carolina C. da Fonseca, Eduardo Tejera, Yunierkis Perez-Castillo, Bruno K. Robbs, Damião P. de Sousa

**Affiliations:** 1 Laboratory of Pharmaceutical Chemistry, Department of Pharmaceutical Sciences, Federal University of Paraíba, Cidade Universitária, João Pessoa 58051-900, Brazil; 2 Postgraduate Program in Applied Science for Health Products, Faculty of Pharmacy, Fluminense Federal University, Niteroi 24241-000, Brazil; 3 Postgraduate Program in Dentistry, Health Institute of Nova Friburgo, Fluminense Federal University, Nova Friburgo 28625-650, Brazil; 4 Bio-Cheminformatics Research Group, Universidad de Las Américas, Quito 170516, Ecuador; 5 Facultad de Ingeniería y Ciencias Aplicadas, Área de Ciencias Aplicadas, Universidad de Las Américas, Quito 170516, Ecuador; 6 Departamento de Ciência Básica, Instituto de Saúde de Nova Friburgo, Universidade Federal Fluminense, Nova Friburgo 28625-650, Brazil

**Keywords:** piplartine, natural products, medicinal plants, anticancer, *Piper*, cytotoxicity, antitumor, alkaloid

## Abstract

Cancer is a principal cause of death in the world, and providing a better quality of life and reducing mortality through effective pharmacological treatment remains a challenge. Among malignant tumor types, squamous cell carcinoma-esophageal cancer (EC) is usually located in the mouth, with approximately 90% located mainly on the tongue and floor of the mouth. Piplartine is an alkamide found in certain species of the genus *Piper* and presents many pharmacological properties including antitumor activity. In the present study, the cytotoxic potential of a collection of piplartine analogs against human oral SCC9 carcinoma cells was evaluated. The analogs were prepared via Fischer esterification reactions, alkyl and aryl halide esterification, and a coupling reaction with PyBOP using the natural compound 3,4,5-trimethoxybenzoic acid as a starting material. The products were structurally characterized using ^1^H and ^13^C nuclear magnetic resonance, infrared spectroscopy, and high-resolution mass spectrometry for the unpublished compounds. The compound 4-methoxy-benzyl 3,4,5-trimethoxybenzoate (**9**) presented an IC_50_ of 46.21 µM, high selectively (SI > 16), and caused apoptosis in SCC9 cancer cells. The molecular modeling study suggested a multi-target mechanism of action for the antitumor activity of compound **9** with CRM1 as the main target receptor.

## 1. Introduction

Cancer is triggered by uncontrolled cell growth through cell differentiation, which can spread to different parts of the body (NIH, 2022). It is considered a global public health problem, and since one in seven deaths in the world is caused by cancer, it is becoming more common than death from HIV/AIDS, tuberculosis, and malaria combined [1].

According to the National Cancer Institute (INCA), 6 million premature deaths are forecast for 2025, with an estimated 1.5 million annual deaths from cancer that might be prevented with adequate measures [2].

Cancers are a great cause of fear in the general population. Lung, prostate, stomach, liver, colorectal, and oral cavity cancers are considered the most common in men, while in women, breast, lung, cervix, colorectal, thyroid, and stomach cancers are more common [3,4,5]. Squamous cell carcinoma-esophageal cancer (EC) is a type of malignant tumor usually located in the mouth, and it represents about 90% of cancers in the tongue and floor of the mouth. The pathology is considered a principal cause of morbidity and mortality worldwide since it is a highly complex and very aggressive cancer that presents a high probability of evolution towards both locoregional and distant metastasis [6,7,8,9].

In research involving medicinal plants, certain species of the Piperaceae family have become the subject of chemical-pharmacological studies due to their potential as a source of bioactive secondary metabolites, especially against tumor cells [10,11,12]. According to Costalotufo et al., the substance piplartine, found in some species of the genus *Piper*, has a trimethoxylated phenylpropanoid structure and can be classified as an alkamide. Piplartine is also known as piperlongumine [13].

The present study aimed to investigate the cytotoxic potential of a collection of piplartine analogs against SCC9 tumor cells derived from oral squamous cell carcinoma. For structure–activity relationships and evaluation of the results obtained in the biological tests, parameters such as lipophilicity, steric effects, and electronic effects were considered for each compound.

## 2. Results

### 2.1. Chemistry

**Scheme 1:**
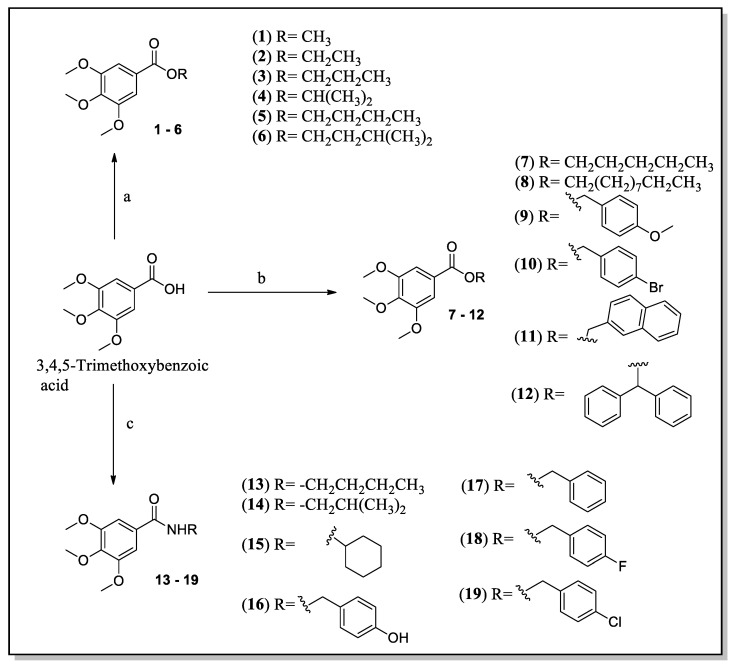
Preparation of **1**–**19**. Reaction conditions: (**a**) ROH, H_2_SO_4_, reflux; (**b**) halide, Et_3_N, acetone, reflux; (**c**) RNH_2_, PyBOP, Et_3_N, CH_2_Cl_2_, 0 °C to r.t.

Preparation of the compounds is demonstrated in Scheme 1. The compounds were prepared by Fischer esterification (a); esterification using alkyl or aryl halides (b); and amidation reactions via the PyBOP coupling agent (c).

The ^1^H and ^13^C NMR spectra of the products synthesized displayed comparable patterns whose signal multiplicity was in accordance with the proposed structures. For the hydrogen signals obtained, two aromatic hydrogens were observed as a singlet ranging from δ _H_ 7.20 to 7.36 ppm and methyl shifts from methoxyls appeared as a singlet ranging from δ _H_ 3.89 to 4.06 ppm. For the ^13^C NMR spectral data, the common signals obtained were signals ranging from δc 165.7 to 166.7 ppm of ester C=O, a signal ranging from δc 152.8 to 153.0 ppm attributed to two carbons, a signal at approximately δc 142.0 ppm, a signal ranging from δc 125.2 to 126.9 ppm, a signal ranging from δc 106.7 to 107.0 ppm for two carbons, a signal from δc 60.8 to 61.0 ppm assigned to a carbon referring to one of the methoxyls, and another signal ranging from δc 56.0 to 56.4 ppm assigned to two carbons from two other methoxyls present in the benzoic ring. For the unpublished esters **6**, **9**, **10**, **11**, and **12**, as well as for the amides **18** and **19**, the described common signals were obtained in addition to other signals for each molecule.

According to the analyses obtained from IR spectroscopy, the following common signals were obtained for esters **1**–**12** above 3000 cm^−1^ (C-H sp2 stretch), while for aromatics, it was signals in pairs at 1600 and 1475 cm^−1^, signals characteristic of an ester-conjugated carbonyl in the range 1740–1715 (C=O), two bands in 1300 to 1000 cm^−1^ (C-O stretch), and range from 1250 to 1040 cm^−1^ (aryl ether stretch) referring to methoxyls. Regarding the amides, the characteristic common signals were a band at approximately 3300 cm^−1^ (-NH) and a band in the range of 1680–1630 cm^−1^ (C=O stretching). Furthermore, the chemical structures were confirmed by HRMS (FT-ICR) spectrometry in accordance with their molecular weights.

### 2.2. Cytotoxicity

#### 2.2.1. Cell Viability (Cytotoxicity) Assay

To determine the new compounds’ cytotoxicity, the ester and amide collections derived from 3,4,5-trimethoxybenzoic acid were first tested on SCC9 cells and fibroblasts by the MTT cell viability assay. Initial screening was performed exclusively in SCC9 cancer cells, as they exhibit a lower stringent phenotype (higher sensitivity) to cytotoxic agents than other SCC cell lines, as previously demonstrated [14]. In addition, DMSO was used as a negative control, and Carboplatin, which is a chemotherapy drug widely used in the clinic for the treatment of this type of cancer, was used as a positive control.

Nonlinear regression curves of viability were generated in relation to the concentration of treatment used to calculate the IC_50_ of each compound (Table 1). Only compounds with IC_50_ lower than 500 μM were tested in fibroblasts to select compounds cytotoxic enough to be useful in future pre-clinical studies and to avoid crystallization at higher concentrations needed to calculate the IC_50_ and selectivity in normal fibroblasts. Compounds that did not reach IC_50_ below 1000 μM were considered non-determined (N.D.; Table 1) for the same reason.

Different species of the genus *Piper* have the alkamide piplartine, which has several biological activities, such as antitumor, cytotoxic, antinociceptive, antiplatelet, and antimetastatic activity [15]. Although piplartine was not tested in this study, the compounds tested are analogous to this molecule. The most active compounds that fit the requisites in SCC9 cells were **3** (IC_50_ = 299 ± 0.06), **4** (373.1 ± 0.05), **5** (204.2 ± 0.07), **6** (160.2 ± 0.07), **7** (257.6 ± 0.08), **9** (46.21 ± 0.16), and **13** (468.8 ± 0.07). Thus, normal fibroblasts were treated with the selected compounds and with Carboplatin to determine selectivity (Table 1). Of these compounds selected for testing on non-tumor cells (normal primary oral fibroblasts), only **7** was not selective (I.S. < 2); others showed intermediary selectivity, as in **5** and **6** (I.S. between 2 and 3), some were highly selective (I.S. > 4), and **9** was the most selective with an S.I. of 16.02. Compounds that present an S.I. ≥ 3 are considered highly selective, because they are three times more cytotoxic to the neoplastic lineage compared to the normal cell and therefore have a large therapeutic window [16]. It is worth noting that **9** was one of the novel compounds and had a 6.5-fold higher selectivity than Carboplatin (S.I. = 2.46), which is the drug of choice in the clinic.

#### 2.2.2. Selectivity

To further characterize the cytotoxicity of the most selective compounds (S.I. > 4), we used another OSCC cell line, SCC4 (Table 2). Of these compounds, only **4** (IC_50_ = 454.3 ± 0.04) and **9** (IC_50_ = 49.81 ± 0.3) showed cytotoxic effects in SCC4 cells. Compound **9** was even more cytotoxic than Carboplatin (IC_50_ = 175.2 ± 0.2) in this tumor cell, with a similar result to SCC9, maintaining its high selectivity (S.I. = 15.42).

The selectiveness of the new compound **9** was further tested against other tumor cell lines from different origins, including B16-F10 (melanoma), HEP-G2 (hepatocarcinoma), and HT-29 (colon adenocarcinoma) (Table 3). Surprisingly, whereas the B16-F10 cell line was found to be more resistant to **9** treatment, HEP G2 and HT-29 were extremely sensitive, with an IC_50_ < 10 µM and S.I > 100.

#### 2.2.3. Hemolytic Activity

**Figure 1:**
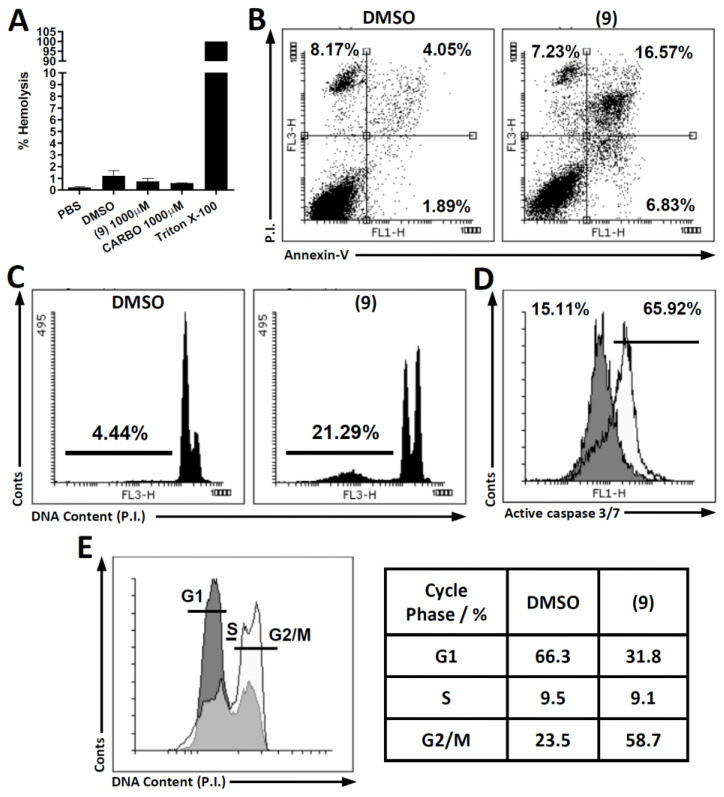
(**A**) Hemolytic activity of compounds at 500 μM (approximately 10 × IC_50_). One-way ANOVA with Dunnett’s post-test was performed where all columns were significantly different from the control (Triton X) with *p* < 0.0001. (**B**) Analysis of phosphatidylserine exposure 48 h after plating. Cells were stained with annexin-V conjugated to FITC and P.I. and were analyzed by flow cytometry. (**C**) Analysis of cell death by propidium iodide (PI) staining. After 48 h of treatment, cells were trypsinized and stained with propidium iodide in the presence of NP-40. The percentage of cell death (SubG0 DNA content) is indicated. (**D**) Flow cytometry data representative of caspase 3/7 activity. SCC9 cells were treated with the equivalent of 2 × IC_50_ of compound **9,** and DMSO was used as a control for 48 h of treatment. The activity of caspase 3/7 of the cells increased significantly under **9** treatment compared to the control. (**E**) Differences in cell cycle distribution. Cell cycle distribution was analyzed after propidium iodide staining and FACS analyses. SCC9 lineage cells were plated in a six-well plate (5 × 10^5^ cells/well). The phases G1, S, and G2 of the cell cycle were classified based on the DNA content after staining with propidium iodide (PI). (**A**–**E**) Results are representative of data from at least three independent experiments.

Since compound **9** had the highest cytotoxicity and selectivity against all cancer cells tested, we further analyzed its tolerability for future in vivo assays by discarding any surfactant activity of the compound which could lead to unspecific cytotoxicity through cellular membrane damage by hemolytic assays. Compound **9** displayed no significant hemolysis (Figure 1A; lower than 1%) in human erythrocytes even in a very high concentration (1000 µM); hemolysis levels were comparable to the controls (carboplatin and DMSO) [17].

#### 2.2.4. Investigation of Cell Death Pathway

In order to elucidate the compound-**9**-induced cell death pathway, flow cytometry assays were performed to analyze apoptotic phenotypes (phosphatidylserine exposition, DNA fragmentation, and caspase 3/7 activation) and cell cycle distribution (Figure 1B–E). Compound **9** showed a four-fold increase (~17% versus ~4% in control) in annexin V single- and/or double-positive (PI^+^) labeling at 48 h (Figure 1B), which is indicative of greater apoptotic processes compared to the control. Compound **9** was also able to induce DNA fragmentation (~21%), as shown by increased SubG1 DNA content (Figure 1C), and a massive executer caspases 3/7 activation (~66%; Figure 1D) when compared to the control (~4% SubG1 and ~15% active caspases), supporting a classic apoptotic phenotype. Thus, our results suggest that **9** induces a classical apoptotic cell death pathway.

Furthermore, treatment of the SCC9 tumor cell with **9** led to a substantial increase in the number of cells exhibiting a G2/M DNA amount compared to the DMSO control (Figure 1E; ~59% × ~23%, respectively).

Together these results demonstrate that the piplartine analogue **9** is highly cytotoxic and selective against different types of cancers possibly through induction of cell cycle arrest at the G2/M phase and an intense cell death induction through apoptosis.

### 2.3. Molecular Modeling

Molecular modeling studies were performed with the main objective of identifying probable molecular targets of compound **9** that could explain its antitumoral activity. The consensus target prediction found 453 potential targets for compound **9** in Homo sapiens. However, we were interested in targets related to cancer. Therefore, the top scored 100 protein candidates (around 22% of the total) were selected for further analysis. From these 100 proteins ACHE, AHR, PTPN1, PPARA, and THRA are driver genes according to the DriverDBv3 database (ranked at positions 5, 10, 53, 63, and 88 respectively). Only the gene MCL1 (at position 57 in the ranking) was classified as a cancer target in the CTD^2^ database. The protein–protein interaction network with direct interactions built from the 89 CTD^2^ targets led to 2355 proteins. From these proteins, MTNR1B, RELA, AHR, NR2F2, MMP2, RIPK2, DUSP3, CASP9, MMP9, PDE4D, RAC1, PTPN7, PTPN1, HDAC6, PDGFRA, MCL1, PPARG, PPARA, PPARD, IMPDH2, PRKCE, CDC42, DNMT1, CYP17A1, NR2E3, THRA, CYP1A2, PTPN2, and HSD17B1 were also present in the consensus prediction at positions: 6, 7, 10, 18, 22, 23, 27, 31, 38, 41, 42, 51, 52, 54, 55, 57, 63, 66, 68, 71, 74, 76, 78, 79, 81, 88, 89, 96, and 98, respectively. Considering the most relevant findings, the possible targets selected for further computational models were AHR, ACHE, THRA, PTPN1, PPARA, MTNR1B, RELA, NR2F2, MMP2, RIPK2, DUSP3, CASP9, and MCL1.

Driver genes can have negative, positive, or ambiguous regulation regarding tumorigenesis or cell proliferation, and individual analyses are required. PTPN1 inhibition has been ambiguous in tumorigenesis (acting as positive or negative regulator) [18,19,20]. A similar situation was found for PPARA [21]. Moreover, THRA (thyroid hormone receptor alpha) has been studied in thyroid, breast, and ovarian cancer [22,23]. However, the resulting protective effect from its direct inhibition is not as clear as for the beta type receptor [24]. On the other hand, ACHE and CASP9 have a protective effect against tumor progression [25,26]. Therefore, these proteins were not selected for further modelling.

Table 4 summarizes the potential targets of compound **9** that were selected from the computational target fishing analyses and from the scientific literature that could explain its antitumoral activity. The table includes the UniProt accession of each target, the IDs employed along the manuscript, a brief functional description, and the source of the structure employed for modeling. The structures of the receptors were mainly obtained from the Protein Data Bank (PDB) database with the exceptions of STAT3 and AHR. In the case of AHR, no structure was available, and a homology model was generated. On the other hand, there were X-ray structures deposited in the PDB for STAT3, but these lack an important segment in the C-terminal region important for ligand binding. For this reason, the AlphaFold model was selected for modeling STAT3.

Molecular docking was performed as described in the Materials and Methods section. The full results of the docking calculations are provided as Supplementary Materials in Table S1. In total, the 13 investigated targets lead to 33 ligand–receptor complexes to be further investigated. In all cases, these 33 complexes showed the compound within the binding sites. The weakest points of docking algorithms are the large approximations required in scoring functions and the limited exploration of the complex’s conformational space. One of the alternatives to overcome these limitations is the post-processing of docking solutions with more accurate and computationally intensive methods based on Molecular Dynamics (MD) simulations. MD-based methods have aided in the identification of the molecular targets of bioactive compounds [27,28], while the estimation of free energies of binding from MD trajectories are effective at discriminating between correct and incorrect ligand-binding poses [29]. According to this evidence, the final selection of the most probable targets of compound **9** was carried out using the MD simulations protocol presented in the Materials and Methods section.

The length of the MD simulations required for predicting the free energy of binding of a ligand to a protein is a topic highly discussed in the scientific literature. Usually, the length of MD simulations is directly related to the available hardware resources. Additionally, there is no consensus on the optimal length of MD simulations required to obtain a conformational ensemble for MM-PBSA calculations. Different authors have concluded that short (fewer than 5 ns) simulations would be sufficient for MM-PBSA calculations [30,31]. Another aspect considered in the literature is that when short MD simulations are used for MM-PBSA calculations, a multiple trajectories approach is preferred over a single trajectory one. Thus, we performed five short MD simulations per complex, each one lasting for 4 ns, and the free energies of binding were estimated from an ensemble of 100 MD snapshots evenly extracted from all five trajectories. With this approach, the total MD simulation time performed in the current research was 660 ns.

The conformational stability of the complexes during MD simulations was measured as the RMSD of the ligand relative to the starting docking pose along each of the 5 ns MD simulations. These plots are provided as Supplementary Materials in Figures S1–S33. Overall, in most of the simulations, the ligand deviated less than or around 2 Å from the starting docking pose during the 4 ns MD simulation time. In no case did the observed RMSD values increase beyond 3 Å. This indicates that the obtained MD trajectories were conformationally stable. Furthermore, the differences in the RMSD plots obtained for a target suggest that a complex explores different conformational spaces during each MD replica.

**Figure 2:**
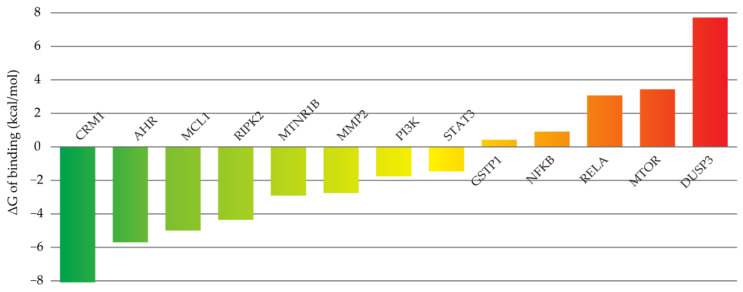
Predicted free energies of binding of compound **9** to its potential targets obtained from the MD simulations. The bars indicate the estimated error of the computed free energies of binding.

The predicted free energies of binding obtained from the MD simulations are given as Supplementary Materials in Table S2 and summarized in Figure 2. Only the top-scored ligand conformer per target is represented in the figure. According to these results, it can be concluded that the most probable target of the compound is CRM1, followed by AHR, MCL1, and RIPK2. This suggests a multi-target mechanism of action for the antitumoral activity of the compound, with CRM1 as the principal receptor for compound **9**. Consequently, the predicted binding mode of the ligand to CRM1 was analyzed in detail.

**Figure 3:**
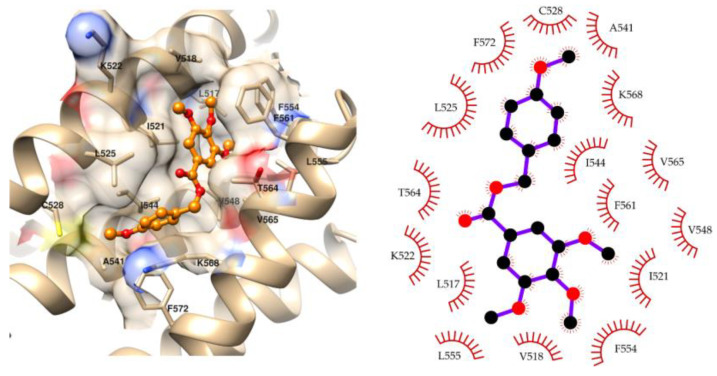
Predicted binding mode and ligand-receptor interactions for the compound **9**–CRM1 complex. The ligand is represented as orange balls and sticks. The receptor and its surface are colored as carbon: tan, nitrogen: blue, oxygen: red, and sulfur: yellow. The figure was produced with UCSF Chimera [35] and LigPlot+ [36].

Figure 3 represents the predicted pose of compound **9** in the binding cavity of CRM1 as well as the ligand–receptor interactions. The complex structure selected for representation was the centroid of the most populated cluster obtained from clustering the MD snapshots used for MM-PBSA calculations. The ligand was predicted to bind in the known mainly hydrophobic groove of CRM1 responsible for the recognition of the nuclear export signals in cargo proteins [32]. Among the 21 HEAT repeats that form CRM1, the inhibitors’ binding cleft was at the interface of repeats 11 and 12.

Specifically, the methoxy of the para-methoxyphenyl group was predicted to orientate toward C528. This is a key residue that is involved in the covalent binding of many known CRM1 inhibitors [32,33]; however, non-covalent inhibitors of this protein have also been reported [34]. The rest of this ring is predicted to interact with L525, A541, I544, K568, and F572. The central linker of the compound interacts with L525, T564, and V565. On the other hand, the trimethoxyphenyl moiety is predicted to occupy a cavity lined by L517, V518, I521, V548, F554, L555, and F561, positioning favorably to form π- π stacking interactions with F561.

Our modeling results were consistent with the observed antitumoral activity of compound **9**. CRM1 is a validated molecular target for anticancer compounds [37,38,39]. This protein is usually overexpressed in cancer and is classified as an oncogenic driver. Specifically, piperlongumine has been shown to be an inhibitor of CRM1 with antitumoral properties [40]. The predicted interaction of compound **9** with C528 is in agreement with the previously observed direct interaction of piperlongumine with this amino acid in CRM1 [40]. The models described here will aid in directing future research focusing on improvement of the antitumoral and CRM1 inhibitory properties of piperlongumine derivatives.

Finally, the ADMET properties of compound **9** were predicted as described in the Materials and Methods section. The results of these predictions are given in Table 5, and they show that compound **9** contains no PAINS alert. In addition, it has favorable physicochemical properties for oral bioavailability. Compound **9** was also predicted to be soluble in water with high gastrointestinal absorption, to be skin permeable, and to not be a substrate of the P-gp. It was predicted to poorly distribute to the brain and to be an inhibitor of three isoforms of the Cytochrome P450. This last aspect, along with its predicted mutagenic potential, should be fully clarified with additional experiments during the hit optimization phase of this compound.

## 3. Discussion

Two ester derivatives (compounds **2** and **10**) presented no cytotoxic activity against SCC9 cells. The others presented low to satisfactory cytotoxicity. In general, the esters were more promising than the amides. However, the starting material, 3,4,5-trimethoxybenzoic acid was inactive.

The alkyl derivatives with medium carbon chains were more potent than the aryl derivatives, except for 4-methoxy-benzyl trimethoxybenzoate (**9**), which was the most potent analog. In fact, compound **9** was 4.5 times more potent and 6.5 times more selective than Carboplatin, the positive control. Carboplatin is used clinically against various types of cancer, such as uterine-cervix cancer, lung carcinoma, gynecological cancers, breast cancer, and others [41,42,43].

It was evident that reducing the size of the carbonic chain reduced the cytotoxic activity for alkyl derivative compounds **4**, **5**, and **6**. However, when comparing straight-chain alkyl derivative propyl 3,4,5-trimethoxybenzoate (**3**) with isopropyl 3,4,5-trimethoxybenzoate (**4**), which is a branched-chain alkyl derivative, greater potency was noted for compound (**3**). N-butyl-3,4,5-trimethoxybenzoamide (**13**) was the only bioactive derivative, yet comparison with butyl 3,4,5-trimethoxybenzoate (**5**) revealed that the ester was 2.3 times more potent. Derivative compound **5** was equipotent to the reference drug. Isopentyl 3,4,5-trimethoxybenzoate (compound **6**) was slightly more potent than compound **5**, and 2-methylnaphthalene 3,4,5-trimethoxybenzoate (**11**) presented greater potency and selectivity than diphenyl 3,4,5-trimethoxybenzoate (**12**), revealing that the fused (naphthalene) ring system potentiates cytotoxic activity.

Fan et al. (2019) [44] evaluated piplartine (10 µM) cytotoxic effects against HepG2 and SMMC-7721 cells from hepatocellular carcinoma and demonstrated that piplartine could be used to eradicate cancers of this type.

Turkez et al. (2019) [45] evaluated a similar piplartine derivative against glioblastoma cancer cells. The molecule presented satisfactory results, and it displayed structural similarity with an amide N-(4-fluorobenzyl)-3,4,5-trimethoxybenzamide (18) prepared in the present study. However, against SCC9 cells, compound **18** was inactive. This suggests that the molecule should be tested against other types of cancer.

The results obtained in the present study corroborate the results obtained by Chen and collaborators (2018) [46], who evaluated piplartine against SAS and CGHNC8 cells, which are oral cancer cell lines. It was shown that piplartine suppressed tumor sphere formation in the oral cancer cells tested, suppressed expression of the SOX2, Oct-4, and NANOG genes associated with stem cells, inhibited cell migration and invasion, and regulated expression of molecules associated with epithelial–mesenchymal transition (EMT). It also increased chemo- and radiosensitivity while even inhibiting tumorigenesis in vitro and in vivo.

Compared to other chemotherapies and to new putative compounds for treatment of OSCC cells, the selectivity results for the tested derivatives were significantly high [47,48,49,50].

Comparing the results obtained in the present study with data from the literature reveals similar cytotoxicities (IC_50_) for piplartine and other analogs against various tumor cells lines, but none demonstrated a higher selectivity than the results displayed by compound **9** [51,52,53].

Test derivative (**9**) results (as well as those of piplartine) did not reveal hemolytic activity in mouse erythrocytes, suggesting that the cytotoxicity of this compound class does not involve membrane damage [17].

Based on the cell death pathway trials, compound **9** is likely involved in apoptotic processes. The derivative was able to induce DNA fragmentation. A similar apoptotic phenotype was observed in PC-3 cells treated with piplartine, with accumulation of fragmented DNA (SubG1) and activation of caspase 3 [54]. Cell cycle arrest in the G2/M phase causes a delay in cell proliferation [55], and this arrest in G2/M has been observed in PC-3 and V79 cells treated with piplartine [54,56].

## 4. Materials and Methods

All reagents necessary for chemical synthesis were obtained from Sigma-Aldrich. Adsorption column chromatography (CC) was performed using silica gel 60, ART 7734 from MERCK as the stationary phase (particle sizes between 0.063 and 0.200 mm). The nuclear magnetic resonance (^1^HNMR and ^13^CNMR) spectra were recorded in Bruker Avance III HD spectrometers operating at 400 MHz (^1^H) and 100 MHz (^13^C) and with a Varian NMR operating at 500 MHz (^1^H) and 125 MHz (^13^C). Chemical shifts were expressed in parts per million (ppm), and the multiplicities of the ^1^HNMR bands were indicated according to the conventions: *s* (singlet), *d* (doublet), *t* (triplet), *quint* (quintet), *sext* (sext), *sept* (septet), and *m* (multiplet). The Fourier Transform Infrared Spectroscopy (FTIR) spectra were obtained using an Agilent technologies Cary 630 FTIR instrument, in the 4000–400 cm^−1^ spectral range. High-resolution mass spectra were obtained at the Biomolecules Mass Spectrometry Center (CEMBIO) of the Health Sciences Center (CCS/UFRJ). The spectrometer used was Solarix XR—FT-ICR configuration (Bruker), with an external temperature with 100 µM sodium formate in water/acetonitrile 1:1 and electrospray ionization source (ESI—nebulizer pressure: 1 bar, 4, 0 L/min, temperature 200 °C, flow rate 4 µL/min), positive mode, detection window 75 to 1200 m/z. The theoretical resolution was 99,000 for m/z 400. The samples were solubilized in 1 mL of methanol and then diluted 1000× in water:acetonitrile 1:1 and injected directly into the mass spectrometer. Melting points were determined in a Microquímica apparatus (Microquímica equipamentos LTDA, Model MQAPF 302, Serial No.: 403/18, Palhoça, Brazil) with temperature measurements in the 10 °C to 350 °C range. All reaction processes were monitored by analytical thin layer chromatography using silica gel sheet chromatographs (Merck 60 F 254).

### 4.1. Preparation of Compounds ***1***–***6***

Sulfuric acid (0.2 mL) was added slowly to a solution containing 3,4,5-trimethoxybenzoic acid (0.1 g; 0.47 mmol) solubilized in 20 mL of alcohol. The reaction mixture was refluxed with magnetic stirring for 5–24 h. After alcohol rotary evaporation, distilled water (10 mL) was added and extraction was performed with dichloromethane (3 × 10 mL). The organic phase was neutralized with 5% sodium bicarbonate (3 × 10 mL), treated with 10 mL of water, and dried with anhydrous sodium sulfate. After purification using column chromatography with silica gel 60 and a mixture of hexane and ethyl acetate in an increasing polarity gradient, yields ranging from 45.3 to 99.6% were obtained.

### 4.2. Preparation of Compounds ***7***–***12***

In a solution containing 3,4,5-trimethoxybenzoic acid 1 Eq. solubilized in 15 mL of anhydrous acetone, 4 Eq. of triethylamine, and 1 Eq. of the halide were added. The reaction mixture was refluxed with magnetic stirring for 72 h. After acetone rotary evaporation, distilled water (10 mL) was added and extraction was performed with dichloromethane (3 × 10 mL). The organic phase was treated with 10 mL of distilled water and dried with anhydrous sodium sulfate. After purification, using column chromatography with silica gel 60 and a mixture of hexane and ethyl acetate in an increasing gradient of polarity, yields ranging from 40.6 to 75.2% were obtained.

### 4.3. Preparation of Compounds ***13***–***19***

A solution containing 3,4,5-trimethoxybenzoic acid (0.1 g; 0.47 mmol) and 0.47 mmol of the corresponding amine solubilized in dimethylformamide (1.0 mL; 0.94 mmol) in the presence of triethylamine (0.06 mL; 0.47 mmol) at 0 °C was prepared. At 30 min, PyBOP coupling agent 0.24 g (0.47 mmol) was added, which had previously been dissolved in dichloromethane (1.0 mL; 0.94 mmol) with magnetic stirring. The reaction time was 6–24 h. After alcohol rotary evaporation, distilled water (10 mL) was added and extraction was performed with dichloromethane (3 × 10 mL). The organic phase was treated with 10% hydrochloric acid (3 × 10 mL) and then neutralized with 5% sodium bicarbonate (3 × 10 mL), treated with 10 mL of distilled water, and dried with anhydrous sodium sulfate. Yields ranging from 44.4 to 91.3% were obtained and purified using column chromatography with silica gel 60 and a mixture of hexane and ethyl acetate in the proportions of 6:4 or 3:7.

Methyl 3,4,5–trimethoxybenzoate (**1**): White crystalline solid; yield 94.9% (101.2 mg; 0.44 mmol); M.P.: 81–83 °C; TLC (8:2 Hexane/AcOEt), R*f* = 0.46; IR ʋmax (KBr, cm^−1^): 3021, 2953; 1716, 1674, 1592 and 1467, 1338 and 1132, 1229 and 992, 863; ^1^H NMR (400 MHz, CDCl_3_,): δ 7.29 (*s*, 2H); 3.89 (*s*, 12H). ^13^C NMR (100 MHz, CDCl_3_): δ 166.59; 152.85; 142.34; 125.26; 106.86; 60.89; 56.23; 52.19 [57].

Ethyl 3,4,5–trimethoxybenzoate (**2**): White solid; yield 94.6% (107.1 mg; 0.44 mmol); M.P.: 53–54 °C; TLC (8:2 Hexane/AcOEt), R*f* = 0.52; IR ʋmax (KBr, cm^−1^): 3014, 2964, 1706, 1664, 1591 and 1456, 1332 and 1132, 1228 and 1042, 863; ^1^H NMR (400 MHz, CDCl_3_,): δ 7.29 (*s*, 2H); 4.39 (*q*, *J* = 7.1 Hz, 2H); 3.90 (*s*, 6H); 3.89 (*s*, 3H); 1.39 (*t*, *J* = 7.1 Hz, 3H); ^13^C NMR (100 MHz, CDCl_3_): δ 166.2); 152.91; 142.09; 125.54; 106.77; 61.13 ; 60.91; 56.24; 14.42 [53].

Propyl 3,4,5–trimethoxybenzoate (**3**): White crystalline solid; yield 99.1% (118.8 mg; 0.46 mmol); M.P.: 34–35 °C; TLC (8:2 Hexane/AcOEt), R*f* = 0.56; IR ʋmax (KBr, cm^−1^): 3114, 2964, 1706, 1664, 1590 and 1459, 1333 and 1124, 1227 and 1008, 858; ^1^H NMR (400 MHz, CDCl_3_): δ 7.30 (*s*, 2H); 4.27 (*t*, *J* = 6.7 Hz, 2H); 3.93 (*s*, 9H); 1.79 (*sext*, *J* = 7.4 Hz, 2H); 1.02 (*t*, *J* = 7.4 Hz, 3H). ^13^C NMR (100 MHz, CDCl_3_): δ 166.14; 152.66; 142.06; 125.46; 106.67; 66.64; 60.95; 56.18; 22.05; 10.44 [57].

Isopropyl 3,4,5–trimethoxybenzoate (**4**): Light brown oil; yield 57.3% (68.6 mg; 0.26 mmol); TLC (8:2 Hexane/AcOEt), R*f* = 0.56; IR ʋmax (KBr, cm^−1^): 3024, 2981, 1711, 1679, 1590 and 1461, 1327 and 1129, 1229 and 1007, 865; ^1^H NMR (400 MHz, CDCl_3_): δ 7.28 (*s*, 2H); 5.24 (*sept*, *J* = 2.2 Hz, 1H); 3.90 (*s*, 6H); 3.89 (*s*, 3H); 1.36 (*d*, *J* = 6.3 Hz, 6H); ^13^C NMR (100 MHz, CDCl_3_): δ 165.74; 152.84; 142.03; 125.91; 106.76; 68.59; 60.91; 56.23; 21.9 [57].

Butyl 3,4,5–trimethoxybenzoate (**5**): Colorless oil; yield 99.6% (125.9 mg; 0.46 mmol); TLC (8:2 Hexane/AcOEt), R*f* = 0.64; IR ʋmax (KBr, cm^−1^): 3006, 2961, 1716, 1655, 1590 and 1459, 1335 and 1129, 1225 and 1006, 865; ^1^H NMR (500 MHz, CDCl_3_): δ 7.29 (*s*, 2H); 4.31 (*t*, *J* = 6.7 Hz, 2H); 3.90 (*s*, 6H); 3.90 (s, 3H); 1.76 (*quint*, *J* = 6.7 Hz, 2H); 1.47 (*sex*, *J* = 7.4 Hz, 2H); 0.98 (*t*, *J* = 7.3 Hz, 3H); ^13^C NMR (125 MHz, CDCl_3_): δ 166.4); 153.04; 142.26; 125.69; 106.96; 65.19; 61.04; 56.40; 30.97; 19.47; 13.91 [53].

Isopentyl 3,4,5–trimethoxybenzoate (**6**): Brown oil, yield 45.3% (60.3 mg; 0.21 mmol); TLC (8:2 Hexane/AcOEt), R*f* = 0.60; IR ʋmax (KBr, cm^−1^): 3002, 2959, 1717, 1655, 1590 and 1459, 1334 and 1129, 1225 and 1006, 865; ^1^H NMR (400 MHz, CDCl_3_): δ 7.29 (*s*, 2H); 4.34 (*t*, *J* = 6.8 Hz, 2H); 3.90 (*s*, 9H); 1.81–1.71 (*m*, 1H); 1.66 (*q*, *J* = 6.8 Hz, 2H); 0.98 (*d*, *J* = 6.6 Hz, 6H); ^13^C NMR (100 MHz, CDCl_3_): δ 166.57; 153.03; 142.29; 125.75; 107.00; 64.13; 61.05; 56.41; 37.62; 25.49; 22.65. HRMS (FT-ICR) analyze: C_15_H_22_O_5_ calculated theoretical value [M+H]+: 283.1540. Found = 283.1538.

Pentyl 3,4,5–trimethoxybenzoate (**7**): White oil; yield 75.2% (100.1 mg; 0.35 mmol); TLC (8:2 Hexane/AcOEt), R*f* = 0.60; IR ʋmax (KBr, cm^−1^): 3012, 2957, 1714, 1657, 1590 and 1461, 1335 and 1129, 1226 and 1006, 865; ^1^H NMR (400 MHz, CDCl_3_): δ 7.29 (*s*, 2H); 4.29 (*t*, *J* = 6.8 Hz, 2H); 3.89 (*s*, 9H); 1.75 (*quint*, *J* = 7.2 Hz, 2H); 1.44–1.34 (*m*, 4H); 0.92 (*t*, *J* = 7.0 Hz, 3H); ^13^C NMR (100 MHz, CDCl_3_): δ 166.27 (C=O); 152.93 (C-3, C-5); 142.13 (C-4); 125.57 (C-1); 106.80; 65.30; 60.96; 56.23; 28.42; 28.18; 22.36; 13.99 [53].

Decyl 3,4,5–trimethoxybenzoate (**8**): Amorphous solid, yield 40.6% (67.4 mg; 0.19 mmol); M.P.: 49–50 °C; TLC (8:2 Hexane/AcOEt), R*f* = 0.64; IR ʋmax (KBr, cm^−1^): 3015, 2956, 1709, 1672, 1590 and 1465, 1336 and 1131, 1226 and 990, 864; ^1^H NMR (400 MHz, CDCl_3_): δ 7.29 (*s*, 2H); 4.30 (*t, J* = 6.8 Hz, 2H); 3.90 (*s*, 9H); 1.80 (*quint*, *J* = 6.8 Hz, 2H); 1.42–1.26 (*m*, 14H); 0.87 (*t*, *J* = 7.0 Hz, 3H); ^13^C NMR (100 MHz, CDCl_3_): δ 166.23; 152.59; 142.05; 125.43; 106.46; 65.33; 60.81; 56.23; 31.89; 29.54; 29.31; 29.28; 28.75; 26.07; 22.71; 14.10 [58].

4-Methoxy-benzyl 3,4,5–trimethoxybenzoate (**9**): White cristalline solid, yield 67.5% (105.7 mg; 0.32 mmol); M.P.: 83–84 °C; TLC (8:2 Hexane/AcOEt), R*f* = 0.36; IR ʋmax (KBr, cm^−1^): 3008, 2944, 1711, 1670, 1589 and 1465, 1332 and 1126, 1228 and 1005, 864; ^1^H NMR (400 MHz, CDCl_3_): δ 7.38 *(d*, *J* = 9.5 Hz, 2H); 7.31 (*s*, 2H, H-2); 6.91 (*d*, *J* = 8.8 Hz, 2H); 5.29 (*s*, 2H,); 3.89 (*s*, 9H); 3.81 (*s*, 3H); ^13^C NMR (100 MHz, CDCl_3_): δ 166.25; 159.66; 152.82, 142.12; 130.01; 128.19; 125.24; 113.89 ; 106.90; 66.69; 60.94; 56.14; 55.27. HRMS (FT-ICR) analyze: C_18_H_20_O_6_ calculated theoretical value [M+H]+: 333.1332. Found = 333.1331.

4-Bromo-benzyl 3,4,5–trimethoxybenzoate (**10**): White cristalline solid, yield 55.0% (197.0 mg; 0.51 mmol); M.P.: 104–105 °C; TLC (8:2 Hexane/AcOEt), R*f* = 0.62; IR ʋmax (KBr, cm^−1^): 3034, 2958, 1712, 1664, 1594 and 1454, 1334 and 1133, 1228 and 1010, 1070, 801; ^1^H NMR (500 MHz, CDCl_3_): δ 7.51 (*d*, *J* = 8.4 Hz, 2H); 7.32 (*d*, *J* = 10 Hz, 2H); 7.31 (s, 2H); 5.30 (s, 2H); 3.90 (*s*, 9H,); ^13^C NMR (125 MHz, CDCl_3_): δ 166.10; 153.12; 142.68; 135.26; 131.93; 130.04; 125.00; 122.50; 107.17; 66.16; 61.10; 56.43. HRMS (FT-ICR) analyze: C_17_H_17_O_5_Br calculated theoretical value [M+H]+: 381.0332. Found = 381.0330.

2-Methylnaphthalene 3,4,5–trimethoxybenzoate (**11**): White solid; yield 48.4% (160.9 mg; 0.45 mmol); M.P.: 84–85 °C; TLC (8:2 Hexane/AcOEt), R*f* = 0.58; IR ʋmax (KBr, cm^−1^): 3002, 2936, 1714, 1670, 1590 and 1463, 1329 and 1129, 1225 and 1008, 864; ^1^H NMR (500 MHz, CDCl_3_): δ 7.91–7.85 (*m*, 4H); 7.55 (*dd*, *J* = 8.4 Hz; 1.6 Hz, 1H), 7.53–7.45 (m, 2H); 7.36 (s, 2H); 5,53 (s, 2H); 3.90 (*s*, 9H); ^13^C NMR (125 MHz, CDCl_3_): δ 166.26; 153.14; 142.55; 133.64; 133.34; 133.29; 128.57; 128.12; 127.85; 127.57; 126.47; 126.44; 126.05; 125.25; 107.18; 67.14; 61.04; 56.41. HRMS (FT-ICR) analyze: C_21_H_20_O_5_ calculated theoretical value [M+H]+: 353.1383. Found = 353.1381.

Diphenyl 3,4,5–trimethoxybenzoate (**12**): Yellow solid; yield 41.5% (148.2 mg; 0.39 mmol); M.P.: 57–58 °C; TLC (8:2 Hexane/AcOEt), R*f* = 0.64; IR ʋmax (KBr, cm^−1^): 3026, 2939, 1727, 1655, 1587 and 1456, 1338 and 1172, 1226 and 1127, 855; ^1^H NMR (500 MHz, CDCl_3_,): δ 7.59–7.47 (m, 12H); 7.44 (s, 1H); 4.06 (*s*, 9H); ^13^C NMR (125 MHz, CDCl_3_,): δ 165.29; 152.96; 142.55; 140.19; 128.58*; 128.47*; 127.99^#^; 127.53 ^#^; 127.15; 126.53; 125.17; 107.15; 77.63; 60.93; 56.31. HRMS (FT-ICR) analyze: C_23_H_22_O_5_ calculated theoretical value [M+H]+: 379.1540. Found= 379.1512.

*N*-Butyl-3,4,5–trimethoxybenzamide (**13**): White solid; yield 89.1% (112.2 mg; 0.41 mmol M.P.: 115–116 °C; TLC (6:4 Hexane/AcOEt), R*f* = 0.36; IR ʋmax (KBr, cm^−1^): 3294, 3017, 2932, 1681, 1634, 1583 and 1459, 1541 and 1506, 1236 and 1131, 843; ^1^H NMR (400 MHz, CDCl_3_): δ 6.98 (*s*, 2H); 6.22 (*s*, 1H); 3.87 (*s*, 6H); 3.86 (*s*, 3H); 3.43 (*q*, *J* = 5.8 Hz, 2H); 1.62 (*quint*, *J* = 7.2 Hz, 2H); 1.38 (*sex*, *J* = 7.5 Hz, 2H); 0.94 (*t*, *J* = 7.3 Hz, 3H); ^13^C NMR (100 MHz, CDCl_3_): δ 167.32; 153.21; 140.87; 130.53; 104.46; 60.97; 56.28; 40.03; 31.91; 20.19; 13.91 [59].

*N*-Isobutyl-3,4,5–trimethoxybenzamide (**14**): Yellow solid; yield 91.3% (115.0 mg; 0.43 mmol); M.P.: 118–119 °C; TLC (6:4 Hexane/AcOEt), R*f* = 0.36 ; IR ʋmax (KBr, cm^−1^): 3307, 3015, 2955, 1687, 1634, 1583 and 1469, 1543 and 1504, 1237 and 1131, 842; ^1^H NMR (500 MHz, CDCl_3_,): δ 6.98 (*s*, 2H), 6.30 (*s*, 1H), 3.86 (*s*, 6H), 3.85 (*s*, 3H); 3.24 (*t*, *J* = 6.5 Hz, 2H), 1.92–1.84 (m, 1H), 0.95 (d, *J* = 6.7 Hz, 6H). ^13^C NMR (125 MHz, CDCl_3_,): δ 167.49; 153.27; 140.75; 130.45; 104.46; 60.94; 56.41; 47.59; 28.74; 20.25 [59].

*N*-Cyclohexyl-3,4,5–trimethoxybenzamide (**15**): White crystalline solid; yield 44.4% (61.4 mg; 0.21 mmol); M.P.: 179–180 °C; TLC (6:4 Hexane/AcOEt), R*f* = 2.2; IR ʋmax (KBr, cm^−1^): 3468, 3077, 2871, 1677, 1621, 1582 and 1463, 1510 and 1417, 1239 and 1004, 844; ^1^H NMR (500 MHz, DMSO-d_6_): δ 7.16 (*s*, 1H); 6.19 (*s*, 2H); 2.86 (*s*, 6H); 2.72 (*s*, 3H); 1.54 (*quint J* = 1.8 Hz, 1H); 0.88–0.76 (*m*, 4H); 0.67–0.62 (*m*, 2H); 0.36-0.32 (*m*, 4H); ^13^C NMR (125 MHz, DMSO-d_6_): δ 164.91; 152.50; 139.87; 130.09; 104.94; 60.22; 56.10; 48.64; 32.63; 25.41; 25.15 [60].

*N*-4-Hydroxybenzyl-3,4,5–trimethoxybenzamide (**16**): White solid, yield 57.15% (256.3 mg; 0.81 mmol); M.P.: 227–229 °C; TLC (5:5 Hexane/AcOEt), R*f* = 0.37; IR ʋmax (KBr, cm^−1^): 3379 and 3314, 3346, 3019, 2099, 1634, 1611, 1574 and 1449, 1545 and 1499, 1414 and 1231, 1211 and 1122, 823; ^1^H NMR (400 MHz, DMSO-d_6_): δ 8,40 (s, 1H); 7.99 (*t*, *J* = 5.7 Hz, 1H,); 6.35 (*s*, 2H); 6.24 (*d*, *J* = 8.5 Hz, 2H); 5.82 (*d*, *J* = 10.0 Hz, 2H); 3.48 (*d*, *J* = 5.7 Hz, 2H); 2.93 (s, 6H); 2.81 (s, 3H); ^13^C NMR (100 MHz, DMSO-d_6_): δ 165.44; 156.32; 152.65; 139.90; 129.89; 129.61; 128.68; 115.06; 104.84; 60.10; 56.01; 42.30 [60].

*N*-Benzyl-3,4,5–trimethoxybenzamide (**17**): White crystalline solid, yield 59.6% (84.6 mg; 0.27 mmol); M.P.: 138–139 °C; TLC (6:4 Hexane/AcOEt), R*f* = 0.38; IR ʋmax (KBr, cm^−1^): 3305, 3028, 2942, 1655, 1625, 1580 and 1459, 1528 and 1499, 1237 and 1127, 840; ^1^H NMR (400 MHz, CDCl_3_): δ 7.35–7.27 (*m*, 5H); 7.03 (*s*, 2H); 6.60 (s, 1H); 4.61 (*d*, *J* = 5,8 Hz, 2H); 3.86 (*s*, 3H); 3.85 (*s*, 6H); ^13^C NMR (100 MHz, CDCl_3_): δ 167.11; 153.34; 141.10; 138.22; 129.87; 128.86; 128.01; 127.70; 104.55; 61.04; 56.34; 44.30 [53].

*N*-(4-Fluorobenzyl)-3,4,5–trimethoxybenzamide (**18**): White crystalline solid, yield 62.5% (90 mg; 0.28 mmol); M.P.: 131–132 °C; TLC (6:4 Hexane/AcOEt), R*f* = 0.34; IR ʋmax (KBr, cm^−1^): 3288, 3012, 2947, 1672, 1634, 1585 and 1459, 1545 and 1508, 1280 and 1130, 1219 and 1098, 827; ^1^H NMR (400 MHz, CDCl_3_): δ 7.29–7.27 (*m*, 2H); 7.01 (*s*, 2H); 6.98 (*d*, *J* = 8,7 Hz, 2H); 6.69 (s, 1H); 4.55 (*d*, *J* = 5,8 Hz, 2H); 3.85 (*s*, 6H); 3.84 (*s*, 3H); ^13^C NMR (100 MHz, CDCl_3_): δ 167.15; 163.50; 161.04; 153.28; 141.17; 134.05; 129.69; 129.66*; 129.58*; 115.74^#^; 115.52^#^; 104.55; 60.90; 56.29; 43.48. HRMS (FT-ICR) analyze: C_17_H_18_FNO_4_ calculated theoretical value [M+H]+: 320.1292. Found = 320.1290.
*, ^#^ Interchangeable.

*N*-4-Chlorobenzyl-3,4,5–trimethoxybenzamide (**19**): White solid; yield 86.6% (137.0 mg; 0.41 mmol); M.P.: 157–158 °C; TLC (6:4 Hexane/AcOEt), R*f* = 0.34; IR ʋmax (KBr, cm^−1^): 3254, 3004, 2946, 1653, 1629, 1582 and 1457, 1538 and 1498, 1235 and 1128, 1070 and 997, 816; ^1^H NMR (500 MHz, CDCl_3_): δ 7.31 (*d*, *J* = 6.4 Hz, 2H); 7.28 (*d*, *J* = 6.4 Hz, 2H); 7.04 (*s*, 2H); 6.74 (s, 1H); 4.58 (*d*, *J* = 5,7 Hz, 2H); 3.88 (*s*, 3H); 3.87 (*s*, 6H); ^13^C NMR (125 MHz, CDCl_3_): δ 167.30; 153.17; 141.26; 136.97; 133.37; 129.60; 129.27; 128.92; 104.58; 61.01; 56.43; 43.55. HRMS (FT-ICR) analyze: C_17_H_18_ClNO_4_ calculated theoretical value [M+H]+: 336.0997. Found =336.0995.

### 4.4. Cytotoxic Activity

#### 4.4.1. Cell Viability (Cytotoxicity) Assay

SCC cells (SCC9 and SCC4), derived from human tongue OSCC, were obtained by (ATCC^®^ CRL-1629 and CRL-1624) and maintained in DMEM/F12 1:1 medium (Dulbecco’s modified Eagle’s medium and Ham’s F12 medium, Gibco (Thermo Fisher, Waltham, MA, USA), supplemented with 10% (*v*/*v*) FBS (Fetal Bovine Serum; Invitrogen, Thermo Fisher, Waltham, MA, USA) and 400 ng/mL hydrocortisone (Sigma-AldrichCo., St. Louis, MO, USA). Primary normal gingival fibroblast human (PCS201 018TM) and other tumor cells B16-F10 (CRL-6475), Hep G2 (HB-8065), and HT-29 (HTB-38) were also obtained at ATCC and maintained in DMEM supplemented with 10% (*v*/*v*) FCS. Cells were cultured in a humidified environment containing 5% CO_2_ at 37 °C. For all experiments, the compounds were solubilized in DMSO (Sigma-Aldrich) for a final concentration of 100 mM. From this dilution, other concentrations were made to carry out the cell viability assay. Carboplatin (Fauldcarbo^®^; Libbs Farmacêutica, São Paulo, SP, Brazil) was used as a control antitumor compound.

The viability of cancer cells and primary fibroblasts was tested using the MTT assay as described in MACHADO et al., 2021 [49]. Briefly, cancer cells (5 × 10^3^ cells/well) were plated in duplicate in a 96-well plate, as indicated. DMSO was diluted in culture medium at the same concentrations and was used as a negative control, representing 100% cell viability. After 48 h of treatment, cells were incubated for 3.5 h with 5 mg/mL of MTT reagent (3,4,5-dimethiazol-2,5-diphenyltetrazoliumbromide) (Sigma-Aldrich Co., St. Louis, MO, USA) diluted in culture medium. Subsequently, the formazan crystals were dissolved by MTT solvent (50% Methanol and 50% DMSO), and the absorbance was read at 560 nm using the EPOCH microplate spectrophotometer (BioTekInstruments, Winooski, VT, USA).

#### 4.4.2. Hemolysis Assay

Human blood was used as approved by the Research Ethics Committee of the Fluminense Federal University–Nova Friburgo, RJ (CAAE: 43134721.4.0000.5626). The erythrocytes were collected by centrifugation at 1500 rpm for 15 min, washed with PBS (phosphate buffer saline) and 10 mM glucose, and counted in an automatic cells counter (Thermo Fisher, Waltham, MA, USA). The erythrocytes were then plated in 96-well plates at a concentration of 4 × 10^8^/well in triplicate, and 10 µL of the compounds were added at a final concentration of 1000 µM in PBS with glucose (final volume of 100 µL). Ten microliters of PBS were used as negative control and 10 µL of PBS with 0.1% Triton ×100 as a positive control. Data reading was performed with EPOCH (BioTek Instruments, Winooski, VT, USA) at 540 nm absorbance, and statistical data were generated with the GRAPHPAD Prism 5.0 program (Intuitive Software for Science, San Diego, CA, USA).

#### 4.4.3. Cell Cycle and SubG1 Analysis

To evaluate the action of the compound **9** on the cell cycle, cells of the SCC9 lineage were plated in a six-well plate (5 × 10^5^ cells/well) as described in LUCENA et al., 2016. After 48h of treatment, cells were trypsinized and stained with propidium iodide (75 µM; Sigma) in the presence of NP-40 (Sigma). DNA content was analyzed by collecting 10,000 events using a FACScalibur flow cytometer. Data were analyzed using CellQuest (BD Biosciences) and FlowJo (Tree Star Inc., Ashland, OR, USA) software. (Version 9.9.4).

#### 4.4.4. Phosphatidylserine Exposure Analysis (Apoptosis)

Cells of the SCC9 cell line were plated in six-well plates (5 × 10^5^ cells/well), trypsinized 48 h after treatment, labeled using the Annexin V-FITC Apoptosis Detection Kit according to the manufacturer’s protocol (#BMS500FI/300, Invitrogen), and analyzed by flow cytometry [61].

#### 4.4.5. Caspase Analysis

For active caspase detection, 5 × 10^4^ SCC9 cells were plated in a 24-well plate containing 1 mL of DMEM/F12 with 10% FBS per well. CellEvent™ Caspase-3/7 reagent (#R37111, Invitrogen) was diluted in culture medium according to the manufacturer’s instructions. Twenty-four hours after plating, cells were treated with Caspase-3/7 reagent and 2× IC50 of **9** or DMSO as a control. Cells were analyzed by flow cytometry after 48 h of treatment.

#### 4.4.6. Statistical Analysis, Calculation of IC_50_, and Selectivity Index (SI)

IC_50_ values for the MTT assay were obtained by non-linear regression using GraphPad Prism 5.0 software (GraphPad, San Diego, CA, USA) from at least three independent experiments. Data are presented as means, ± standard deviation (SD). A log dose response (inhibitor) vs. response curve using the least square method was used to determine the IC_50_ and SD of the data. The selectivity index was calculated as: S.I. = IC_50_ of the compound in fibroblasts/IC_50_ of the same compound in tumor cells.

### 4.5. Modeling Study

#### 4.5.1. Target Selection

Targets were selected according to two criteria: a literature survey for the mechanism of action of piperlongumine and a computational target fishing approach. The first strategy lead to the selection of the proteins signal transducer and activator of transcription 3 (STAT3) [62], nuclear factor NF-kappa-B p105 subunit (NFKB1) [63], exportin-1 (CRM1) [40], phosphatidylinositol 4,5-bisphosphate 3-kinase (PIK3) [64], serine/threonine-protein kinase mTOR (mTOR) [64], and glutathione S-transferase P (GSTP1) [65] for modeling studies.

The computational methodology used for target prediction consisted in the consensus of different models of target–compound interaction predictions previously applied in other investigations [66,67]. Briefly, all the possible target proteins of compound **9** in Homo sapiens were predicted using the following algorithms: MolTarPred [68], Swisstarget Prediction [69], Targetnet Scbdd [70], Targetnet Scbdd Ensemble [70], RF QSAR [71], and PPB2 [72]. The following algorithms from PPB2 were employed for predictions: PPB2—Extended Connectivity fingerprint ECfp4 NN, PPB2—Shape and Pharmacophore fingerprint Xfp NN, PPB2—Molecular Quantum Numbers MQN NN, PPB2—Extended Connectivity fingerprint ECfp4 NNNB, PPB2—Shape and Pharmacophore fingerprint Xfp NNNB, PPB2—Molecular Quantum Numbers MQN NNNB, PPB2—Extended Connectivity fingerprint ECfp4 NB, and PPB2—Extended Connectivity fingerprint DNN. Therefore, a total of 13 algorithms were used to predict the possible target proteins of compound **9**. For each target, a final score was computed as:

$$FS_{i}=\sqrt{\bar{F_{i}}\;\frac{N_{i}}{M}}$$

where: $\bar{F_{i}}$ is the average normalized score of target *i* across all methods that predict it, *N* is the number of methods identifying the *i*-th target, and *M* is the number of target fishing algorithms [73] employed.

Usually, many proteins comprising different metabolic pathways or biological processes are found with this consensus strategy. However, only those involved in cancer are relevant for studying the potential antitumoral mechanism of action of compound **9**. To detect possible proteins related to cancer we used two different databases: (1) DriverDBv3 [74] and (2) Cancer Target Discovery and Development (CTD^2^) [75]. The first database is an integrative multi-omics database comprising driver genes organized across different types of cancer. All the 1457 driver genes reported in the database were considered. It is important to notice that a driver gene does not necessarily imply that targeting it will produce an antitumoral activity. The second database is a team effort organized by the National Cancer Institute and the NIH with the aim of validating discoveries from large-scale adult and pediatric cancer-genome research and their use in precision medicine. From this database, 89 genes classified as molecular targets with different evidence levels (Tier 1 to 3) were selected.

It is possible that some of the potential targets identified by the consensus analysis are not necessarily targets for antitumoral compounds (as defined in the CTD^2^ database) or a driver gene (as defined in the DriverDBv3 database). Therefore, we constructed a protein–protein interaction network with the top 89 targets from CTD^2^ restricted to only direct interactions using the BioGrid [76] database and Cytoscape [77]. The top 100 candidate targets obtained from the consensus target fishing approach were filtered to keep only those also present in the list provided by the analysis of the DriverDBv3 and CTD^2^ databases.

#### 4.5.2. Molecular Docking

Molecular docking was performed following the previously described methodology [78,79]. One initial three-dimensional (3D) structure of compound **9** was generated with OpenEye´s Omega [80,81], and partial atomic charges of type am1bcc were added to it with Molcharge [82]. Docking calculations were carried out with Gold [83].

The explored binding sites for compound **9** were defined from the ligands and inhibitors co-crystallized with the target proteins when these were present in their PDB files. This was the case for NFKB (PDB 1SVC), CRM1 (PDB 6TVO), PI3K (PDB 4JPS), mTOR (PDB 4JSP), GSTP1 (PDB 5J41), MTNR1B (PDB 6ME6), MMP2 (PDB 1HOV), RIPK2 (PDB 5J79), DUSP3 (PDB 3F81), and MCL1 (PDB 6UDV). For STAT3, despite using the model available in the AlphaFold repository, the ligand-binding cavity was defined after superimposing this model with an experimental structure of the protein in complex with an inhibitor (PDB 6NJS). In the case of AHR, the antagonist present in one of the model templates identified by the SwissModel server (PDB 4ZQD) was used as a reference for defining the binding cavity after structural superimposition with the homology model. On the other hand, for RELA the ligand-binding region was defined from the segment of DNA interacting with it on the X-ray structure (PDB 3GUT). In all cases, the detect cavity option of Gold was switched on, and the binding region was defined as any receptor residue at a distance lower than 6 Å from the reference ligand.

The ChemPLP scoring function was selected for primary docking, and 30 diverse binding hypotheses were generated for each target. The search efficiency parameter of Gold was set to 200%. The binding modes predicted were rescored with the scoring functions GoldScore, ChemScore, and ASP. Scoring values were converted to Z-scores and aggregated as in our previous publications [78,79]. All ligand conformations with a Z-score value larger than one were selected for further analyses.

#### 4.5.3. Molecular Dynamics Simulations and Free Energies of Binding

Molecular dynamics (MD) simulations were performed with Amber 22 [84] as in our previous reports [79,85]. The ff19SB, gaff2, and lipid17 force fields were used for the parametrization of amino acids, compound **9,** and lipids, respectively. The Zn^2+^ ion and its coordinating residues in MMP2 were prepared according to the Cationic Dummy Atom (CaDA) model [86,87].

Except for the MTNR1B membrane protein, all systems were prepared following the same procedure. All these complexes were enclosed in truncated octahedron boxes that were solvated with OPC water molecules. Excess charges were neutralized by the addition of Na^+^ and Cl^−^ counterions at a concentration of 150 mM following the methodology described in [86]. The solvated systems were energy minimized in two stages with all atoms except those solvent restrained during the first of these. All constraints were released for the second energy minimization step. Energy minimized systems were heated from 0 K to 300 K and subsequently equilibrated at constant temperature and constant pressure.

The complexes with MTNR1B were prepared with the CHARM-GUI server [28,29]. Bilayer membranes containing 50 1-Palmotoyl-2-oleoylglycero-3-phosphocholine (POPC), 50 1-Palmitoyl-2-oleoylphosphatidylethanolamine (POPE), and 25 Cholesterol (CHL) molecules on each side were constructed to embed these systems. Excess charges were neutralized as in the soluble proteins (see above). The energy minimization, heating, and equilibration steps for these complexes were performed using the configuration files provided by CHARM-GUI.

Five short (4 ns) MD simulations were performed for each, including the membrane protein. The atomic initial velocities were randomly initialized for every production run to ensure a diverse exploration of the complexes’ conformational space. Parameters for the production runs were the same for all the systems.

The free energies of binding were estimated using the MM-PBSA methods as implemented in the MMPBSA.py script of Amber 22. These calculations took place over 100 MD snapshots evenly selected from the 1 ns–4 ns time interval each of the five production runs. The ionic strength for MM-PBSA calculations was set to 150 mM. Default implicit solvent parameters were employed for the complexes of compound **9** with soluble proteins. For MTNR1B, a heterogeneous dielectric implicit membrane model (memopt = 3) was employed. The thickness and center of the implicit membrane was defined from average positions of the N31 atoms in the explicit bilayer used for MD simulations.

#### 4.5.4. ADMET Predictions

The absorption, distribution, metabolism, excretion, and toxicology (ADMET) properties of compound **9** were predicted with the SwissADME [88] and pkCSM [89] servers. The prediction of the physicochemical properties as well as the PAINS analysis were performed with SwissADME, while the rest of the predictions were made with the pkCSM server.

## 5. Conclusions

This study presents preparation, cytotoxic evaluation, and molecular modeling of a series of piplartine analogs against tumor cells. SAR revealed the influence of carbon chain size on pharmacological activity. Compound 4-methoxy-benzyl 3,4,5-trimethoxybenzoate (**9**) presented the best cytotoxic profile (IC_50_ = 46.21 µM with a selectivity index of 16.02). Its potency and selectivity were superior to the reference drug (carboplatin). The in silico study suggested that the most likely compound **9** target was CRM1, followed by AHR, MCL1, and RIPK2, suggesting a potential multi-target mechanism of action for its antitumor activity and corroborating the results obtained in the cytotoxicity assays. In accordance with the results obtained in the present study, compound **9** may also be useful in other studies to synthesize analogs in an attempt to optimize its activity. Other compounds derived from 3,4,5-trimethoxybenzoic acid may also be tested against differing cancer cell lines to assess their cytotoxicity. The results of our study may contribute to the development of new antitumor agents.

## Figures and Tables

**Table 1:** IC_50_ results for compounds **1**–**19**.

COMPOUND	R	SCC9		Primary Gingival Fibroblast		S.I.
		IC_50_ μM (µg/mL)	S.D. μM (µg/mL)	IC_50_ μM (µg/mL)	S.D. μM (µg/mL)	
ESTERS						
**1**	-CH_3_	858.5 (194.21)	0.10 (0.022)	-	-	-
**2**	-CH_2_CH_3_	N.D.	-	-	-	-
**3**	-CH_2_CH_2_CH_3_	299 (76.03)	0.06 (0.015)	1256 (319.37)	0.04 (0.010)	4.20
**4**	-CH(CH_3_)_2_	373.1 (94.87)	0.05 (0.013)	1795 (456.43)	0.07 (0.018)	4.81
**5**	-CH_2_CH_2_CH_2_CH_3_	204.2 (54.79)	0.07 (0.019)	510.4 (136.94)	0.04 (0.012)	2.50
**6**	-CH_2_CH_2_CH(CH_3_)_2_	160.2 (45.23)	0.07 (0.2)	569.8 (160.87)	0.07 (0.2)	3.56
**7**	CH_2_CH_2_CH_2_CH_2_CH_3_	257.6 (72.73)	0.08 (0.02)	417.6 (117.9)	0.02 (0.0056)	1.62
**8**	-CH_2_(CH_2_)_7_CH_2_CH_3_	954.9 (336.57)	0.18 (0.063)	-	-	-
**9**	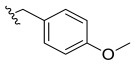	46.21 (15.36)	0.16 (0.053)	740.6 (246.14)	0.03 (0.009)	16.02
**10**	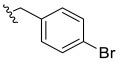	N.D.	-	-	-	
**11**	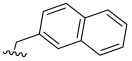	309.9 (109.2)	0.04 (0.014)	3406 (1200.2)	0.06 (0.021)	3.78
**12**	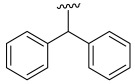	829.8 (314.01)	0.26 (0.098)	-	-	-
AMIDES						
**13**	-CH_2_CH_2_CH_2_CH_3_	468.8 (125.32)	0.07 (0.019)	2359 (630.61)	0.05 (0.013)	5.03
**14**	-CH_2_CH(CH_3_)_2_	N.D.	-	-	-	-
**15**	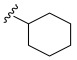	N.D.	-	-	-	-
**16**	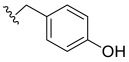	N.D.		-	-	-
**17**	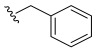	N.D.	-	-	-	-
**18**	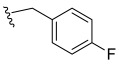	N.D.	-	-	-	-
**19**	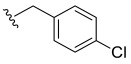	N.D.	-	-	-	-
3,4,5-Trimethoxybenzoic acid	H	N.D.	-	-	-	-
Carboplatin	-	208.4 (77.37)	0.05 (0.018)	512.3 (190.19)	0.02 (0.0074)	2.46

Determination of IC_50_ and selectivity index of collections of esters and amides derived from 3,4,5-trimethoxybenzoic acid. SCC9 (OSCC cells) and normal human gingival fibroblasts were treated with the indicated compounds for 48 h, and cell viability was determined as indicated in materials and methods. Shown from left to right: compound nomenclature, SCC9 tumor or normal fibroblast, IC_50_ (μM), S.D., and selectivity index (S.I.) from at least three independent experiments. S.I. = IC_50_ of the compound in cells of normal oral fibroblasts/IC_50_ of the same compound for oral cancer lineage SCC9. S.D. = standard deviation. N.D. = not determined, not toxic to SCC cells.

**Table 2:** Characterization of the most selective compounds in other OSCC cells.

	Oral Tumor Cells					Primary Gingival Fibroblast		Average S.I.
COMPOUND	SCC9		SCC4		Average			
	IC_50_	S.D.	IC_50_	S.D.		IC_50_	S.D.	
**3**	299.0	0.06	N.D.	N.D.	N.D.	1256	0.04	N.D.
**4**	373.1	0.05	454.3	0.04	375.6	1795	0.07	4.79
**9**	46.21	0.16	49.81	0.34	48.01	740.6	0.03	15.42
**13**	468.8	0.07	N.D.	N.D.	N.D.	2359	0.05	N.D.
CARBOPLATIN	208.4	0.05	175.2	0.15	191.8	512.3	0.02	2.67

The IC_50_ (μM) of two different OSCC cell lines (SCC9 and SCC4) and normal fibroblast cells followed by the average of the selectivity index of compounds **3**, **4**, **9,** and **13** were calculated in the same way as in Table 1. All values are results of at least three independent experiments.

**Table 3:** Selectivity of compound **9**.

Tumor Cells	COMPOUND 9		
	IC_50_	S.D.	S.I.
B16-F10	73.29	0.06	10.10
HEP G2	6.63	0.15	111.7
HT-29	7.31	0.23	101.3

**Table 4:** Potential molecular targets of compound **9**.

UniProt Accession	Target ID ^(a)^	Description	Source ^(b)^
P40763 ^(c)^	STAT3	Signal transducer and activator of transcription 3	AlphaFold
P19838 ^(c)^	NFKB	Nuclear factor NF-kappa-B p105 subunit	PDB (1SVC)
O14980 ^(c)^	CRM1	Exportin-1	PDB (6TVO)
P42336 ^(c)^	PI3K	Phosphatidylinositol 4,5-bisphosphate 3-kinase catalytic subunit alpha isoform	PDB (4JPS)
P42345 ^(c)^	MTOR	Serine/threonine-protein kinase mTOR	PDB (4JSP)
P09211 ^(c)^	GSTP1	Glutathione S-transferase P	PDB (5J41)
P35869 ^(d)^	AHR	Aryl hydrocarbon receptor	SwissModel
P49286 ^(d)^	MTNR1B	Melatonin receptor type 1B	PDB (6ME6)
Q04206 ^(d)^	RELA	Transcription factor p65	PDB (3GUT)
P08253 ^(d)^	MMP2	72 kDa type IV collagenase	PDB (1HOV)
O43353 ^(d)^	RIPK2	Receptor-interacting serine/threonine-protein kinase 2	PDB (5J79)
O43353 ^(d)^	DUSP3	Receptor-interacting serine/threonine-protein kinase 2	PDB (3F81)
Q07820 ^(d)^	MCL1	Induced myeloid leukemia cell differentiation protein Mcl-1	PDB (6UDV)

^(a)^ ID used for the target along the manuscript. ^(b)^ Source of the receptor structure employed for modeling studies. ^(c)^ Protein selected from the scientific literature survey. ^(d)^ Protein derived from the computational target fishing approach.

**Table 5:** Predicted ADMET properties of compound **9**.

Parameter	Compound 9
Physicochemical properties	
Molecular weight (g/mol)	332.35
Rotatable bonds	8
H-bond acceptors	6
H-bond donors	0
Fraction Csp3	0.28
TPSA (A3)	63.22
**Lipophilicity (Log Po/w)**	
iLOGP	3.64
XLOGP3	3.15
MLOGP	2.1
Consensus	3.06
**Absorption**	
Water solubility (log mol/L)	−4.88
GI (%)	97.58
Log Kp (skin permeation) cm/s	−2.74
P-gp substrate	No
**Distribution**	
BBB permeability (log BB)	−0.124
CNS permeation (Log PS)	−3.02
VD (human) (log L/kg)	−0.425
**Metabolism**	
CYP1A2 inhibitor	Yes
CYP2C9 inhibitor	No
CYP2C19 inhibitor	Yes
CYP3A4 inhibitor	Yes
CYP2D6 inhibitor	No
**Excretion**	
Total Clearance (log mL/min/kg)	0.59
Renal OCT2 substrate	No
**Toxicity**	
AMES toxicity	Yes
Max. tolerated dose (human) (log mg/kg/day)	1.44
hERG I inhibitor	No
hERG II inhibitor	No
Oral rat acute toxicity (LD50) (mol/kg)	2.34
Oral rat chronic toxicity (LOAEL) (log mg/kg_bw/day)	1.53
Hepatotoxicity	No
Skin Sensitisation	No

## Data Availability

Not applicable.

## Supplementary Materials

The following supporting information can be downloaded at: https://www.mdpi.com/article/10.3390/molecules28041675/s1, Table S1: Results of docking compound **9** to its potential targets; Table S2: Predicted free energies of binding according to the MM-PBSA method; Figures S1–S33: RMSD of compound **9** relative to its docking pose along the MD simulations.

## Sample Availability

Samples of the compounds are available from the authors.

## List of Abbreviations

AcOEt	ethyl acetate
ADMET	Absorption, Distribution, Metabolism, Excretion and Toxicity
AHR	Aryl hydrocarbon receptor
ATCC	American Type Culture Collection
B16-F10	cell lines from origin of melanoma
CC	column chromatography
CDCl_3_	deuterated chloroform
CRM1	Exportin-1
CTD^2^	Cancer Target Discovery and Development
DMEM	Dulbecco′s Modified Eagle′s Medium
DMSO-*d_6_*	Dimethyl sulfoxide-d_6_
DUSP3	Receptor-interacting serine/threonine-protein kinase 2
EC	Esophageal Cancer
$\bar{F_{i}}$	Average normalized score of target *i*
FITC	Fluorescein isothiocyanate
FS*_i_*	fluid-structure interaction
FTIR	Fourier Transform Infrared Spectroscopy
GSTP1	Glutathione S-transferase P
HRMS	High Resolution Mass spectra
HEP-G2	cell lines from origin of hepatocarcinoma
HT-29	cell lines from origin of colon adenocarcinoma
Hz	Hertz
IC_50_	inhibitory concentration 50%
^1^H NMR	Hydrogen Nuclear Magnetic Resonance
^13^C NMR	Carbon Thirteen Nuclear Magnetic Resonance
INCA	National Cancer Institute
*i*-th	Target
KBr	Potassium bromide
M	Number of target fishing algorithms
MCL1	Induced myeloid leukemia cell differentiation protein Mcl-1
MD	Molecular Dynamics
MD-based	Molecular Dynamics-based
MHz	Megahertz
MMP2	72 kDa type IV collagenase
MP	Melting points
MTOR	Serine/threonine-protein kinase mTOR
MTNR1B	Melatonin receptor type 1B
MTT	3,4,5-dimethiazol-2,5-diphenyltetrazoliumbromide
*N*	Number of methods identifying
N.D.	not determined
NFKB	Nuclear factor NF-kappa-B p105 subunit
NIH	National Institutes of Health
NP-40	Tergitol Type NP-40
OSCC	Oral Squamous Cell Carcinoma
PBS	Phosphate buffered saline
P.I.	propidium iodide
PI3K	Phosphatidylinositol 4,5-bisphosphate 3-kinase catalytic subunit alpha isoform
PyBOP	(Benzotriazol-1-yloxy)tripyrrolidinophosphonium hexafluorophosphate
RELA	Transcription factor p65
R*f*	Retardation factor
RIPK2	72 kDa type IV collagenase
SAR	STRUCTURE ACTIVITY RELATION
SAS	Tongue squamous cell carcinoma cell line.
STAT3	Signal transducer and activator of transcription 3
SCC	Squamous Cell Carcinoma
SCC4	Squamous Cell Carcinoma—4
SCC9	Squamous Cell Carcinoma—9
S.I.	selectivity index
S.D.	standard deviation
TLC	thin layer chromatography
V-FITC	ANNEXIN V-FITC

## References

[1] American Cancer Society (ACS). https://www.cancer.org/content/dam/cancer-org/research/cancer-facts-andstatistics/annual-cancer-facts-and-figures/2017/cancer-facts-and-figures-2017.pdf.

[2] Instituto Nacional de Câncer José Alencar Gomes da Silva. https://www.inca.gov.br/en/node/3776#:~:text=A%20menos%20que%20sejam%20tomadas,ser%20evitadas%20com%20medidas%20adequadas.

[3] Instituto Nacional de Câncer (INCA). http://www.inca.gov.br/estimativa/2014/index.asp?ID=2.

[4] Organização Mundial da Saúde (OMS). http://www.who.int/mediacentre/news/releases/2003/pr27/en/.

[5] Zhang Y., Somtakoune S.D., Cheung C., Listiawan M., Feng X. (2016). Therapeutic Application of Pharmacogenomics in Oncology. AAPS J..

[6] Brener S., Jeunon F.A., Barbosa A.A., Grandinetti H.D.A.M. (2007). Carcinoma De células Escamosas Bucal: Uma revisão De Literatura Entre O Perfil Do Paciente, Estadiamento clínico E Tratamento Proposto. Rev. Bras. Cancerol..

[7] Massano J., Regateiro F.S., Januário G., Ferreira A. (2006). Oral squamous cell carcinoma: Review of prognostic and predictive factors. Oral Surg. Oral Med. Oral Pathol. Oral Radiol. Endodontol..

[8] Blatt S., Ziebart T., Krüger M., Pabst A.M. (2016). Diagnosing oral squamous cell carcinoma: How much imaging do we really need? A review of the current literature. J. Cranio-Maxillofac. Surg..

[9] Feller L., Lemmer J. (2012). Oral Squamous Cell Carcinoma: Epidemiology, Clinical Presentation and Treatment. J. Cancer Ther..

[10] Wang Y.-H., Morris-Natschke S.L., Yang J., Niu H.-M., Long C.-L., Lee K.-H. (2014). Anticancer Principles from Medicinal Piper (胡椒 Hú Jiāo) Plants. J. Tradit. Complement. Med..

[11] Bezerra D.P., Pessoa C., de Moraes M.O., Saker-Neto N., Silveira E.R., Costa-Lotufo L.V. (2013). Overview of the therapeutic potential of piplartine (piperlongumine). Eur. J. Pharm. Sci..

[12] Regasini L.O., Cotinguiba F., Passerini G.D., Bolzani V.D.S., Cicarelli R.M.B., Kato M., Furlan M. (2009). Trypanocidal activity of Piper arboreum and Piper tuberculatum (Piperaceae). Rev. Bras. de Farm..

[13] Costa-Lotufo L.V., Montenegro R.C., Alves A.P., Madeira S.V., Pessoa C., Moraes M.E., Moraes M.O. (2010). A contribuição dos produtos naturais como fonte de novos fármacos anticâncer: Estudos no Laboratório Nacional de Oncologia Experimental da Universidade Federal do Ceará. Rev. Virtual de Quím.

[14] da Fonseca A.C.C., de Queiroz L.N., Felisberto J.S., Ramos Y.J., Marques A.M., Wermelinger G.F., Pontes B., Moreira D.D.L., Robbs B.K. (2020). Cytotoxic effect of pure compounds from *Piper rivinoides* Kunth against oral squamous cell carcinoma. Nat. Prod. Res..

[15] Nóbrega F.R., Silva L.V., Bezerra Filho C.D., Lima T.C., Castillo Y.P., Bezerra D.P., Lima T.K., Sousa D.P. (2019). Design, Antileishmanial Activity, and QSAR Studies of a Series of Piplartine Analogues. J. Chem..

[16] Mahavorasirikul W., Viyanant V., Chaijaroenkul W., Itharat A., Na-Bangchang K. (2010). Cytotoxic activity of Thai medicinal plants against human cholangiocarcinoma, laryngeal and hepatocarcinoma cells in vitro. BMC Complement. Altern. Med..

[17] Bezerra D.P., Pessoa C., de Moraes M.O., Silveira E.R., Lima M.A.S., Elmiro F.J.M., Costa-Lotufo L.V. (2005). Antiproliferative Effects of Two Amides, Piperine and Piplartine, from Piper Species. Z. Naturforsch C J. Biosci..

[18] Bollu L.R., Mazumdar A., Savage M.I., Brown P.H. (2017). Molecular Pathways: Targeting Protein Tyrosine Phosphatases in Cancer. Clin. Cancer Res..

[19] Nunes-Xavier C.E., Aurtenetxe O., Zaldumbide L., López-Almaraz R., Erramuzpe A., Cortés J.M., López J.I., Pulido R. (2019). Protein tyrosine phosphatase PTPN1 modulates cell growth and associates with poor outcome in human neuroblastoma. Diagn. Pathol..

[20] Xu Q., Wu N., Li X., Guo C., Li C., Jiang B., Wang H., Shi D. (2019). Inhibition of PTP1B blocks pancreatic cancer progression by targeting the PKM2/AMPK/mTOC1 pathway. Cell Death Dis..

[21] Tan Y., Wang M., Yang K., Chi T., Liao Z., Wei P. (2021). PPAR-α Modulators as Current and Potential Cancer Treatments. Front. Oncol..

[22] Heublein S., Mayr R., Meindl A., Angele M., Gallwas J., Jeschke U., Ditsch N. (2015). Thyroid Hormone Receptors Predict Prognosis in BRCA1 Associated Breast Cancer in Opposing Ways. PLoS ONE.

[23] Shinderman-Maman E., Cohen K., Moskovich D., Hercbergs A., Werner H., Davis P.J., Ellis M., Ashur-Fabian O. (2017). Thyroid hormones derivatives reduce proliferation and induce cell death and DNA damage in ovarian cancer. Sci. Rep..

[24] Davidson C.D., Gillis N.E., Carr F.E. (2021). Thyroid Hormone Receptor Beta as Tumor Suppressor: Untapped Potential in Treatment and Diagnostics in Solid Tumors. Cancers.

[25] Xi H., Wu R., Liu J., Zhang L., Li Z. (2015). Role of acetylcholinesterase in lung cancer. Thorac. Cancer.

[26] Hakem R., Hakem A., Duncan G.S., Henderson J.T., Woo M., Soengas M.S., Elia A., de la Pompa J.L., Kagi D., Khoo W. (1998). Differential Requirement for Caspase 9 in Apoptotic Pathways In Vivo. Cell.

[27] Wang J., Morin P., Wang W., Kollman P.A. (2001). Use of MM-PBSA in Reproducing the Binding Free Energies to HIV-1 RT of TIBO Derivatives and Predicting the Binding Mode to HIV-1 RT of Efavirenz by Docking and MM-PBSA. J. Am. Chem. Soc..

[28] Katsila T., Spyroulias G.A., Patrinos G.P., Matsoukas M.-T. (2016). Computational approaches in target identification and drug discovery. Comput. Struct. Biotechnol. J..

[29] Guterres H., Im W. (2020). Improving Protein-Ligand Docking Results with High-Throughput Molecular Dynamics Simulations. J. Chem. Inf. Model..

[30] Wang E., Sun H., Wang J., Wang Z., Liu H., Zhang J.Z.H., Hou T. (2019). End-Point Binding Free Energy Calculation with MM/PBSA and MM/GBSA: Strategies and Applications in Drug Design. Chem. Rev..

[31] Poli G., Granchi C., Rizzolio F., Tuccinardi T. (2020). Application of MM-PBSA Methods in Virtual Screening. Molecules.

[32] Shaikhqasem A., Dickmanns A., Neumann P., Ficner R. (2020). Characterization of Inhibition Reveals Distinctive Properties for Human and *Saccharomyces cerevisiae* CRM1. J. Med. Chem..

[33] Etchin J., Sun Q., Kentsis A., Farmer A., Zhang Z.C., Sanda T., Mansour M., Barceló C., McCauley D., Kauffman M. (2012). Antileukemic activity of nuclear export inhibitors that spare normal hematopoietic cells. Leukemia.

[34] Lei Y., An Q., Shen X.-F., Sui M., Li C., Jia D., Luo Y., Sun Q. (2021). Structure-Guided Design of the First Noncovalent Small-Molecule Inhibitor of CRM1. J. Med. Chem..

[35] Pettersen E.F., Goddard T.D., Huang C.C., Couch G.S., Greenblatt D.M., Meng E.C., Ferrin T.E. (2004). UCSF Chimera?A visualization system for exploratory research and analysis. J. Comput. Chem..

[36] Laskowski R.A., Swindells M.B. (2011). LigPlot+: Multiple ligand–protein interaction diagrams for drug discovery. J. Chem. Inf. Model..

[37] Ferreira B.I., Cautain B., Grenho I., Link W. (2020). Small Molecule Inhibitors of CRM1. Front. Pharmacol..

[38] Dickmanns A., Monecke T., Ficner R. (2015). Structural Basis of Targeting the Exportin CRM1 in Cancer. Cells.

[39] Azizian N.G., Li Y. (2020). XPO1-dependent nuclear export as a target for cancer therapy. J. Hematol. Oncol..

[40] Niu M., Xu X., Shen Y., Yao Y., Qiao J., Zhu F., Zeng L., Liu X., Xu K. (2015). Piperlongumine is a novel nuclear export inhibitor with potent anticancer activity. Chem. Interact..

[41] Garces H.I., Mora P.A.R., Alves F.V.G., Carmo C.C.D., Grazziotin R., Fernandes A.C.F.M., Nogueira-Rodrigues A., de Melo A.C. (2013). First-Line Paclitaxel and Carboplatin in Persistent/Recurrent or Advanced Cervical Cancer. Int. J. Gynecol. Cancer.

[42] Avelino C.U.R., Cardoso R.M., De Aguiar S.S., Da Silva M.J.S. (2015). Assessment of quality of life in patients with advanced non-small cell lung carcinoma treated with a combination of carboplatin and paclitaxel. J. Bras. Pneumol..

[43] Díaz-Guardamino I.E. (2017). Identificación de Predictores de Respuesta a Quimioterapia Neodayuvante con Carboplatino-docetaxel de Pacientes con Cáncer de Mama Triple Negative. Ph.D. Thesis.

[44] Fan X., Song J., Zhao Z., Chen M., Tu J., Lu C., Wu F., Zhang D., Weng Q., Zheng L. (2019). Piplartine suppresses proliferation and invasion of hepatocellular carcinoma by LINC01391-modulated Wnt/β-catenin pathway inactivation through ICAT. Cancer Lett..

[45] Turkez H., da Nóbrega F.R., Ozdemir O., Filho C.D.S.M.B., de Almeida R.N., Tejera E., Perez-Castillo Y., de Sousa D.P. (2019). NFBTA: A Potent Cytotoxic Agent against Glioblastoma. Molecules.

[46] Chen Y.G., Satpathy A.T., Chang H.Y. (2017). Gene regulation in the immune system by long noncoding RNAs. Nat. Immunol..

[47] Zorzanelli B.C., de Queiroz L.N., Santos R.M., Menezes L.M., Gomes F.C., Ferreira V.F., Silva F.D.C.D., Robbs B.K. (2018). Potential cytotoxic and selective effect of new benzo[*b*]xanthenes against oral squamous cell carcinoma. Futur. Med. Chem..

[48] Chipoline I.C., da Fonseca A.C.C., da Costa G.R.M., de Souza M.P., Rabelo V.W.-H., de Queiroz L.N., de Souza T.L.F., de Almeida E.C.P., Abreu P.A., Pontes B. (2020). Molecular mechanism of action of new 1,4-naphthoquinones tethered to 1,2,3-1H-triazoles with cytotoxic and selective effect against oral squamous cell carcinoma. Bioorganic Chem..

[49] Machado T.Q., Felisberto J.R.S., Guimarães E.F., de Queiroz G.A., da Fonseca A.C.C., Ramos Y.J., Marques A.M., Moreira D.D.L., Robbs B.K. (2021). Apoptotic effect of β-pinene on oral squamous cell carcinoma as one of the major compounds from essential oil of medicinal plant *Piper rivinoides* Kunth. Nat. Prod. Res..

[50] Zorzanelli B.C., Ouverney G., Pauli F.P., da Fonseca A.C.C., de Almeida E.C.P., de Carvalho D.G., Possik P.A., Rabelo V.W.-H., Abreu P.A., Pontes B. (2022). Pro-Apoptotic Antitumoral Effect of Novel Acridine-Core Naphthoquinone Compounds against Oral Squamous Cell Carcinoma. Molecules.

[51] Bezerra D.P., Militão G.C.G., de Castro F.O., Pessoa C., de Moraes M.O., Silveira E.R., Lima M.A.S., Elmiro F.J.M., Costa-Lotufo L.V. (2007). Piplartine induces inhibition of leukemia cell proliferation triggering both apoptosis and necrosis pathways. Toxicol. In Vitro.

[52] Jyothi D., Vanathi P., Gowri P.M., Rao V.R.S., Rao J.M., Sreedhar A. (2009). Diferuloylmethane augments the cytotoxic effects of piplartine isolated from Piper chaba. Toxicol. In Vitro.

[53] Da Nóbrega F.R., Ozdemir O., Sousa S.C.S.N., Barboza J.N., Turkez H., De Sousa D.P. (2018). Piplartine Analogues and Cytotoxic Evaluation against Glioblastoma. Molecules.

[54] Ahn S.-C., Kong E., Kim Y.-J., Cho H.-J., Yu S.-N., Kim K.-Y., Chang J.-H., Ahn T. (1994). Piplartine induces caspase-mediated apoptosis in PC-3 human prostate cancer cells. Oncol. Rep..

[55] Huang H.-W., Tang J.-Y., Ou-Yang F., Wang H.-R., Guan P.-Y., Huang C.-Y., Chen C.-Y., Hou M.-F., Sheu J.-H., Chang H.-W. (2018). Sinularin Selectively Kills Breast Cancer Cells Showing G2/M Arrest, Apoptosis, and Oxidative DNA Damage. Molecules.

[56] Bezerra D.P., Moura D.J., Rosa R.M., de Vasconcellos M.C., e Silva A.C.R., de Moraes M.O., Silveira E.R., Lima M.A.S., Henriques J.A.P., Costa-Lotufo L.V. (2008). Evaluation of the genotoxicity of piplartine, an alkamide of Piper tuberculatum, in yeast and mammalian V79 cells. Mutat. Res. Toxicol. Environ. Mutagen..

[57] Green R.A., Pletcher D., Leach S.G., Brown R.C.D. (2015). N-Heterocyclic Carbene-Mediated Oxidative Electrosynthesis of Esters in a Microflow Cell. Org. Lett..

[58] Iranpoor N., Firouzabadi H., Riazi A., Pedrood K. (2016). Regioselective hydrocarbonylation of phenylacetylene to α,β-unsaturated esters and thioesters with Fe(CO)5 and Mo(CO)6. J. Organomet. Chem..

[59] Zinchenko A.N., Muzychka L.V., Smolii O., Bdzhola V.G., Protopopov M.V., Yarmoluk S.M. (2017). Synthesis and biological evaluation of novel amino-substituted derivatives of pyrido[2,3-d]pyrimidine as inhibitors of protein kinase CK2. Biopolym. Cell.

[60] Obrecht D., Bohdal U., Broger C., Bur D., Lehmann C., Ruffieux R., Schönholzer P., Spiegler C., Müller K. (1995). L-Phenylalanine Cyclohexylamide: A simple and convenient auxiliary for the synthesis of optically pure α,α-disubstituted (R)- and (S)-amino acids. Helvetica Chim. Acta.

[61] Faget D.V., Lucena P.I., Robbs B.K., Viola J.P.B. (2012). NFAT1 C-Terminal Domains Are Necessary but Not Sufficient for Inducing Cell Death. PLoS ONE.

[62] Yao Y., Sun Y., Shi M., Xia D., Zhao K., Zeng L., Yao R., Zhang Y., Li Z., Niu M. (2016). Piperlongumine induces apoptosis and reduces bortezomib resistance by inhibiting STAT3 in multiple myeloma cells. Oncotarget.

[63] Zheng J., Son D.J., Gu S.M., Woo J.R., Ham Y.W., Lee H.P., Kim W.J., Jung J.K., Hong J.T. (2016). Piperlongumine inhibits lung tumor growth via inhibition of nuclear factor kappa B signaling pathway. Sci. Rep..

[64] Shrivastava S., Kulkarni P., Thummuri D., Jeengar M.K., Naidu V.G.M., Alvala M., Redddy G.B., Ramakrishna S. (2014). Piperlongumine, an alkaloid causes inhibition of PI3 K/Akt/mTOR signaling axis to induce caspase-dependent apoptosis in human triple-negative breast cancer cells. Apoptosis.

[65] Harshbarger W., Gondi S., Ficarro S.B., Hunter J., Udayakumar D., Gurbani D., Singer W.D., Liu Y., Li L., Marto J.A. (2017). Structural and Biochemical Analyses Reveal the Mechanism of Glutathione S-Transferase Pi 1 Inhibition by the Anti-cancer Compound Piperlongumine. J. Biol. Chem..

[66] Tejera E., Pérez-Castillo Y., Toscano G., Noboa A.L., Ochoa-Herrera V., Giampieri F., Álvarez-Suarez J.M. (2021). Computational modeling predicts potential effects of the herbal infusion “horchata” against COVID-19. Food Chem..

[67] Beltrán-Noboa A., Proaño-Ojeda J., Guevara M., Gallo B., Berrueta L.A., Giampieri F., Perez-Castillo Y., Battino M., Álvarez-Suarez J.M., Tejera E. (2022). Metabolomic profile and computational analysis for the identification of the potential anti-inflammatory mechanisms of action of the traditional medicinal plants Ocimum basilicum and Ocimum tenuiflorum. Food Chem. Toxicol..

[68] Peón A., Naulaerts S., Ballester P.J. (2017). Predicting the Reliability of Drug-target Interaction Predictions with Maximum Coverage of Target Space. Sci. Rep..

[69] Daina A., Michielin O., Zoete V. (2019). SwissTargetPrediction: Updated data and new features for efficient prediction of protein targets of small molecules. Nucleic Acids Res..

[70] Yao Z.-J., Dong J., Che Y.-J., Zhu M.-F., Wen M., Wang N.-N., Wang S., Lu A.-P., Cao D.-S. (2016). TargetNet: A web service for predicting potential drug–target interaction profiling via multi-target SAR models. J. Comput. Mol. Des..

[71] Lee K., Lee M., Kim D. (2017). Utilizing random Forest QSAR models with optimized parameters for target identification and its application to target-fishing server. BMC Bioinform..

[72] Awale M., Reymond J.-L. (2018). Polypharmacology Browser PPB2: Target Prediction Combining Nearest Neighbors with Machine Learning. J. Chem. Inf. Model..

[73] Liu S.-H., Shen P.-C., Chen C.-Y., Hsu A.-N., Cho Y.-C., Lai Y.-L., Chen F.-H., Li C.-Y., Wang S.-C., Chen M. (2019). DriverDBv3: A multi-omics database for cancer driver gene research. Nucleic Acids Res..

[74] Aksoy B.A., Dančík V., Smith K., Mazerik J.N., Ji Z., Gross B., Nikolova O., Jaber N., Califano A., Schreiber S.L. (2017). CTD2 Dashboard: A searchable web interface to connect validated results from the Cancer Target Discovery and Development Network. Database.

[75] Oughtred R., Rust J., Chang C., Breitkreutz B., Stark C., Willems A., Boucher L., Leung G., Kolas N., Zhang F. (2020). TheBioGRIDdatabase: A comprehensive biomedical resource of curated protein, genetic, and chemical interactions. Protein Sci..

[76] Shannon P., Markiel A., Ozier O., Baliga N.S., Wang J.T., Ramage D., Amin N., Schwikowski B., Ideker T. (2003). Cytoscape: A software environment for integrated models of Biomolecular Interaction Networks. Genome Res..

[77] Perez-Castillo Y., Lima T.C., Ferreira A.R., Silva C.R., Campos R.S., Neto J.B.A., Magalhães H.I.F., Cavalcanti B.C., Júnior H.V.N., de Sousa D.P. (2020). Bioactivity and Molecular Docking Studies of Derivatives from Cinnamic and Benzoic Acids. BioMed. Res. Int..

[78] Lopes S.P., Castillo Y.P., Monteiro M.L., de Menezes R.R., Almeida R.N., Martins A., Sousa D.P. (2019). Trypanocidal Mechanism of Action and in silico Studies of p-Coumaric Acid Derivatives. Int. J. Mol. Sci..

[79] OMEGA OpenEye Scientific Software, Santa Fe, NM. https://www.eyesopen.com/search?term=OMEGA.

[80] Hawkins P.C.D., Skillman A.G., Warren G.L., Ellingson B.A., Stahl M.T. (2010). Conformer Generation with OMEGA: Algorithm and Validation Using High Quality Structures from the Protein Databank and Cambridge Structural Database. J. Chem. Inf. Model..

[81] QUACPAC OpenEye Scientific Software, Santa Fe, NM. https://www.eyesopen.com/search?term=QUACPAC.

[82] Jones G., Willett P., Glen R.C., Leach A.R., Taylor R. (1997). Development and validation of a genetic algorithm for flexible docking. J. Mol. Biol..

[83] Case D., Aktulga H., Belfon K., Ben-Shalom I., Berryman J., Brozell S., Cerutti D., Cheatham T., Cis-neros G., Cruzeiro V. (2022). AMBER 2022.

[84] Araújo M.O., Pérez-Castillo Y., Oliveira L.H.G., Nunes F.C., De Sousa D.P. (2020). Larvicidal Activity of Cinnamic Acid Derivatives: Investigating Alternative Products for *Aedes aegypti* L. Control. Molecules.

[85] Mayo Clinic Zinc Protein Simulations Using the Cationic Dummy Atom (CaDA) Method. https://www.mayo.edu/research/labs/computer-aided-molecular-design/projects/zinc-protein-simulations-using-cationic-dummy-atom-cada-approach.

[86] Pang Y.P., Xu K., Yazal J.E., Prendergas F.G. (2000). Successful molecular dynamics simulation of the zinc-bound farnesyltransferase using the cationic dummy atom approach. Protein Sci..

[87] Machado M.R., Pantano S. (2020). Split the Charge Difference in Two! A Rule of Thumb for Adding Proper Amounts of Ions in MD Simulations. J. Chem. Theory Comput..

[88] Daina A., Michielin O., Zoete V. (2017). SwissADME: A free web tool to evaluate pharmacokinetics, drug-likeness and medicinal chemistry friendliness of small molecules. Sci. Rep..

[89] Pires D.E.V., Blundell T.L., Ascher D.B. (2015). pkCSM: Predicting Small-Molecule Pharmacokinetic and Toxicity Properties Using Graph-Based Signatures. J. Med. Chem..

