# Supplementary material for: Molecular Modeling and In Vitro Evaluation of Piplartine Analogs against Oral Squamous Cell Carcinoma
Source: Molecules. 2023 Feb 9;28(4):1675. doi: 10.3390/molecules28041675 (PMC9964404; doi:10.3390/molecules28041675)
Supplement: Supplementary file 1 [file molecules-28-01675-s001.zip › molecules-2153827-supplementary.pdf]

Supplementary Materials

Molecular modeling and *in vitro* evaluation of piplartine analogs against oral squamous cell carcinoma

Rayanne H. N. Silva[1], Thaíssa Q. Machado[2], Anna Carolina C. da Fonseca[3], Eduardo Tejera[4], Yunierkis Perez-Castillo[5], Bruno K. Robbs[6,*] and Damião P. de Sousa[1,*]

[1] Laboratory of Pharmaceutical Chemistry, Department of Pharmaceutical Sciences, Federal University of Paraíba, Cidade Universitária, João Pessoa, Paraíba, 58051-900, Brazil

[2] Postgraduate Program in Applied Science for Health Products, Faculty of Pharmacy, Fluminense Federal University, Niteroi, Brazil

[3] Postgraduate Program in Dentistry, Health Institute of Nova Friburgo, Fluminense Federal University, Nova Friburgo, Brazil

[4] Bio-Cheminformatics Research Group. Universidad de Las Américas. Quito 170516. Ecuador

[5] Facultad de Ingeniería y Ciencias Aplicadas. Área de Ciencias Aplicadas. Universidad de Las Américas. Quito 170516. Ecuador

[6] Departamento de Ciência Básica, Instituto de Saúde de Nova Friburgo, Universidade Federal Fluminense, CEP 28625-650, Nova Friburgo-RJ, Brasil

* Correspondence: damiao_desousa@yahoo.com.br or brunokr@id.uff.br

Table S1. *Results of docking compound* ***9*** *to its potential targets.*

Target	Pose	PLP[(a)]	Z_PLP[(b)]	GS[(c)]	Z_GS[(d)]	CS[(e)]	Z_CS[(f)]	ASP[(g)]	Z_ASP[(h)])	Aggregated Z-score
STAT3	1	57.3	0.81	30.87	1.26	21.98	1.43	28.65	2.02	1.38
	2	57.29	0.81	26.19	0.76	23.89	2.01	27.81	1.76	1.33
	3	58.46	1.23	34.51	1.65	20.2	0.88	24.59	0.76	1.13
NFKB	1	55.94	1.32	17.32	0.84	17.94	1.48	27.24	0.92	1.14
	2	57.85	1.66	14.27	0.66	15.57	0.61	30.48	1.48	1.1
	3	58.24	1.72	-2.85	-0.34	18.22	1.58	29.51	1.31	1.07
CRM1	1	68.92	1.96	22.79	0.36	29.78	2.31	35.11	1.97	1.65
	2	68.39	1.82	25.61	0.54	27.96	1.45	34.06	1.6	1.35
	3	69.87	2.2	11.89	-0.31	27.24	1.1	34.74	1.84	1.21
PI3K	1	68.72	2.12	34.86	1.79	25.67	0.96	40.61	1.8	1.67
	2	67.41	1.79	12.13	-0.78	28.34	2.55	37.87	0.94	1.13
	3	67.45	1.8	19.67	0.07	25.67	0.96	39.27	1.38	1.05
	4	65.72	1.37	23.83	0.54	26.88	1.68	36.65	0.55	1.03
MTOR	1	69.98	1.32	13.42	0.27	29.91	1.64	33.3	0.49	0.93
GSTP1	1	41.12	2.17	-231.46	0.46	0.09	1.89	11.14	2.87	1.85
	2	35.28	1.08	-223.56	1.13	-0.94	1.52	4.74	1.11	1.21
AHR	1	64.38	2.69	-14.28	1.15	18.09	1.69	28.46	1.93	1.87
	2	58.6	1.98	5.49	1.73	9.06	0.46	26.16	1.61	1.45
	3	46.37	0.48	-42.14	0.34	21.61	2.16	28.27	1.91	1.22
	4	53.98	1.41	-43.4	0.3	17.96	1.67	23.13	1.19	1.14
MTNR1B	1	61.17	2.38	26.62	1.06	22.84	1.93	31.37	1.42	1.7
	2	54.57	0.84	29.87	1.15	22.55	1.85	30.38	1.2	1.26
RELA	1	52.18	1.48	21.35	0.63	19.11	2.12	24.24	0.28	1.13
MMP2	1	77.9	2.88	6.34	0.68	22.18	2.46	23.78	0.72	1.69
	2	66.64	1.41	-2.93	0.21	18.74	1.48	30.88	2.01	1.28
	3	62.99	0.93	9.88	0.85	20	1.84	22.76	0.53	1.04
RIPK2	1	58.05	1.56	20.9	0.8	24.15	1.06	27.18	1.11	1.13
	2	58.71	1.76	12.6	0.34	25.53	1.52	25.97	0.75	1.09
	3	54.81	0.54	24.24	0.99	23.09	0.71	29.8	1.88	1.03
DUSP3	1	51.3	0.55	11.86	0.77	15.38	0.89	28.74	1.23	0.86
MCL1	1	68.86	2.43	23.09	0.65	28.91	2.63	34.06	1.5	1.8
	2	68.05	2.24	19.85	0.53	26.23	1.51	35.59	1.98	1.56
	3	64.29	1.37	10.84	0.18	28.09	2.29	30.98	0.54	1.09

[(a)] PLP score. [(b)] PLP Z-score. [(c)] GoldScore score. [(d)] GoldScore Z-score. [(e)] ChemScore score. [(f)] ChemScore Z-score. [(g)] ASP score. [(h)] ASP Z-score.

Table S2. *Predicted free energies pf binding of compound **9** to its potential targets and their components.*

Target	Pose	VD WAALS	EEL	EPB	ENPOLAR	EDISPER	ΔG gas	ΔG solv.	ΔG Total	Estimated Error
STAT3	1	-41.41	-10.40	36.40	-32.01	51.50	-51.82	55.88	4.07	0.64
	2	-43.74	-13.63	36.98	-33.14	52.07	-57.37	55.91	-1.46	0.58
	3	-41.85	-10.24	33.86	-31.40	50.39	-52.09	52.84	0.76	0.52
NFKB	1	-36.28	-15.41	39.37	-26.39	41.42	-51.69	54.41	2.71	0.54
	2	-30.82	-12.66	31.37	-21.99	35.01	-43.48	44.39	0.91	0.35
	3	-24.77	-13.34	28.15	-18.77	29.79	-38.10	39.17	1.07	0.34
CRM1	1	-39.28	-2.34	17.40	-31.78	49.13	-41.61	34.75	-6.87	0.33
	2	-38.64	-3.10	17.62	-31.50	47.92	-41.74	34.04	-7.70	0.37
	3	-39.84	-2.68	17.30	-32.15	49.28	-42.52	34.44	-8.08	0.32
PI3K	1	-34.70	-13.06	29.26	-25.97	43.58	-47.76	46.87	-0.89	0.41
	2	-35.17	-8.96	28.42	-26.83	45.05	-44.12	46.65	2.52	0.38
	3	-35.23	-12.27	28.62	-25.84	42.97	-47.50	45.75	-1.76	0.37
	4	-33.01	-14.71	29.97	-24.56	42.62	-47.72	48.04	0.31	0.40
MTOR	1	-43.47	-15.54	41.43	-32.50	53.51	-59.01	62.44	3.43	0.65
GSTP1	1	-34.30	-9.65	27.10	-25.27	42.55	-43.95	44.38	0.42	0.35
	2	-40.04	-5.41	27.95	-28.56	46.78	-45.45	46.17	0.72	0.38
AHR	1	-51.41	-11.76	35.44	-36.66	58.70	-63.17	57.47	-5.70	0.43
	2	-50.80	-12.43	38.39	-35.18	57.71	-63.23	60.92	-2.30	0.46
	3	-51.00	-11.61	35.97	-36.49	59.19	-62.61	58.67	-3.94	0.44
	4	-52.12	-9.84	35.95	-36.84	59.33	-61.95	58.44	-3.51	0.41
MTNR1B	1	-51.34	25.58	0.00	-36.59	59.45	-25.76	22.86	-2.90	0.45
	2	-49.90	28.41	0.00	-35.90	59.67	-21.49	23.77	2.27	0.41
RELA	1	-21.73	-15.60	29.34	-17.23	28.29	-37.33	40.40	3.07	0.35
MMP2	1	-48.66	-22.34	52.00	-35.10	55.14	-71.00	72.05	1.05	0.48
	2	-43.44	-11.86	39.47	-31.20	50.31	-55.30	58.58	3.28	0.56
	3	-48.58	-26.77	52.37	-34.93	55.15	-75.34	72.59	-2.75	0.63
RIPK2	1	-44.88	-16.96	40.92	-32.79	53.88	-61.83	62.00	0.17	0.50
	2	-46.69	-16.99	39.04	-33.85	54.14	-63.68	59.33	-4.35	0.48
	3	-44.45	-14.15	34.41	-32.80	53.11	-58.60	54.71	-3.89	0.50
DUSP3	1	-27.59	-4.70	25.99	-21.23	35.25	-32.29	40.01	7.72	0.56
MCL1	1	-37.71	-5.36	22.86	-28.81	46.35	-43.07	40.40	-2.67	0.33
	2	-38.17	-3.32	20.60	-27.67	43.57	-41.49	36.50	-4.99	0.42
	3	-43.63	-4.30	24.77	-32.26	51.47	-47.94	43.98	-3.96	0.36

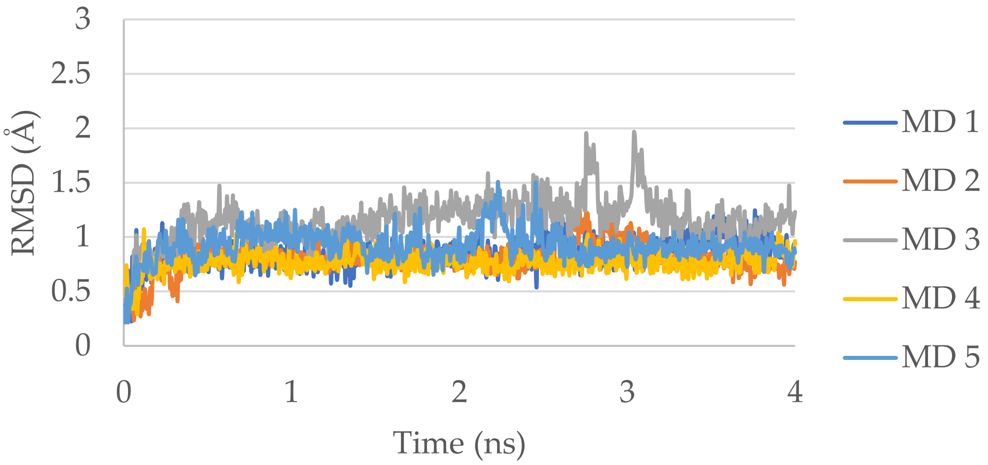

Figure S1. Ligand RMSD vs. time for the AHR-compound **9** complex (docking pose 1).

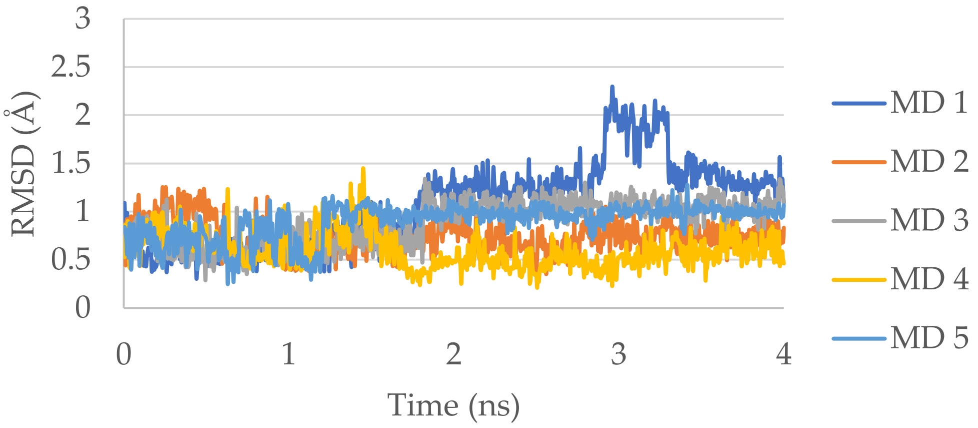

Figure S2. Ligand RMSD vs. time for the AHR-compound **9** complex (docking pose 2).

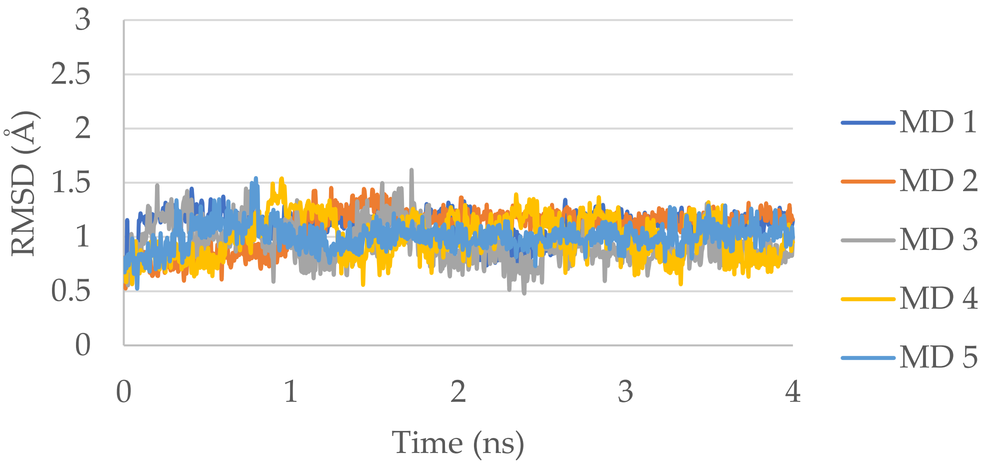

Figure S3. Ligand RMSD vs. time for the AHR-compound **9** complex (docking pose 3).

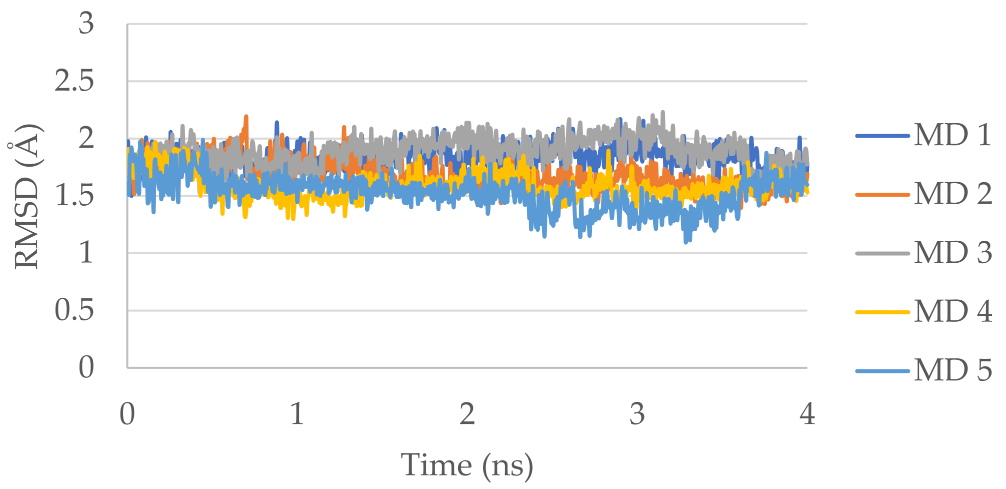

Figure S4. Ligand RMSD vs. time for the AHR-compound **9** complex (docking pose 4).

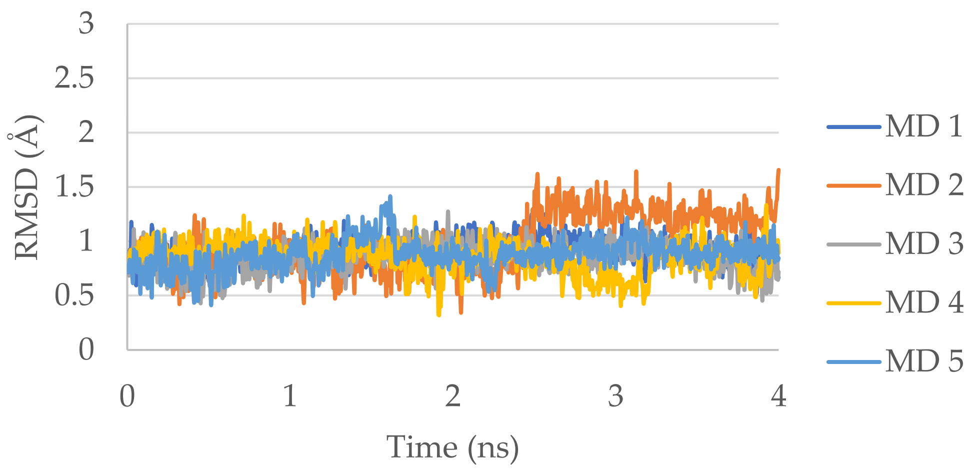

Figure S5. Ligand RMSD vs. time for the CRM1-compound **9** complex (docking pose 1).

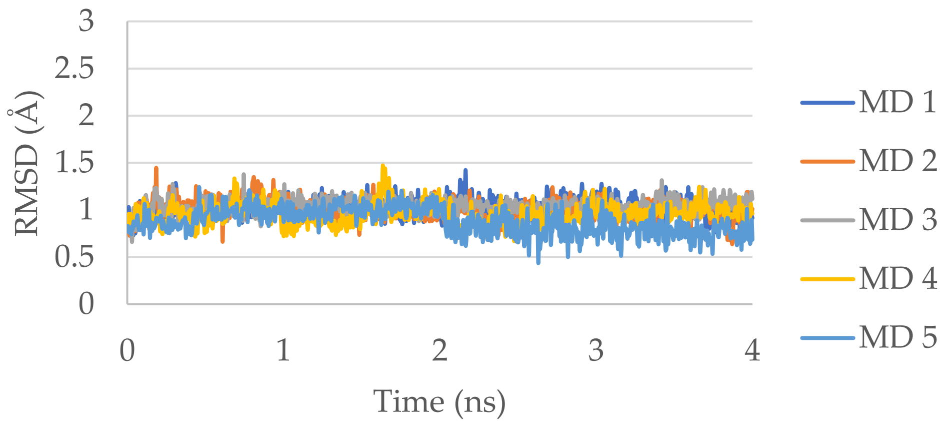

Figure S6. Ligand RMSD vs. time for the CRM1-compound **9** complex (docking pose 2).

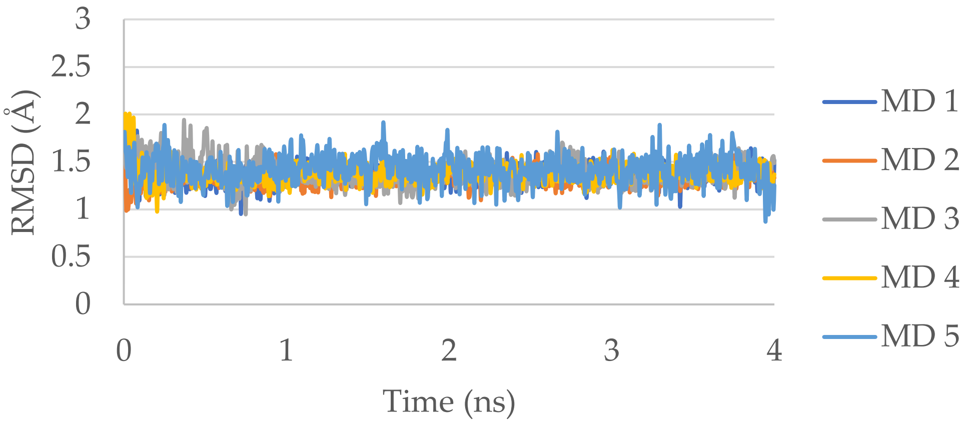

Figure S7. Ligand RMSD vs. time for the CRM1-compound **9** complex (docking pose 3).

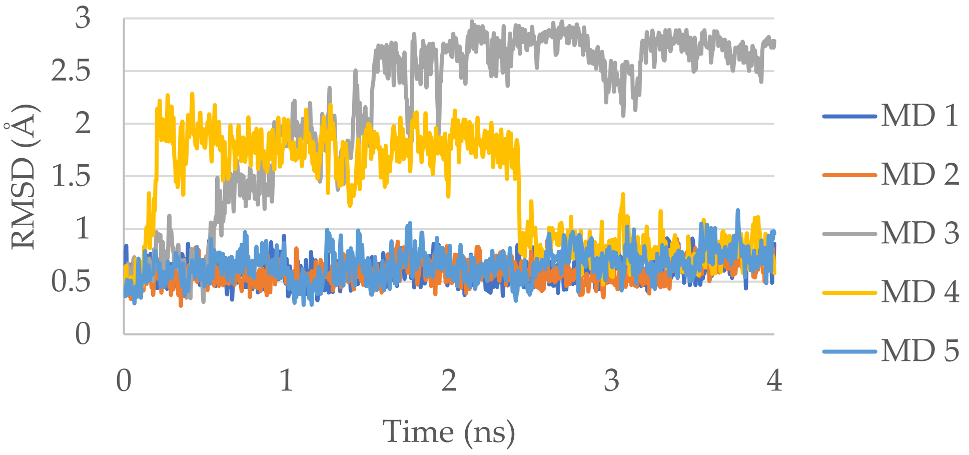

Figure S8. Ligand RMSD vs. time for the DUSP3-compound **9** complex (docking pose 1).

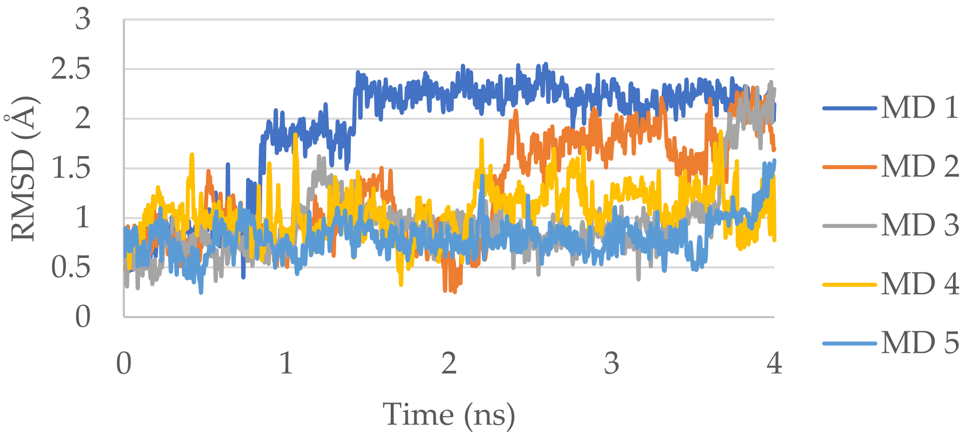

Figure S9. Ligand RMSD vs. time for the GSTP1-compound **9** complex (docking pose 1).

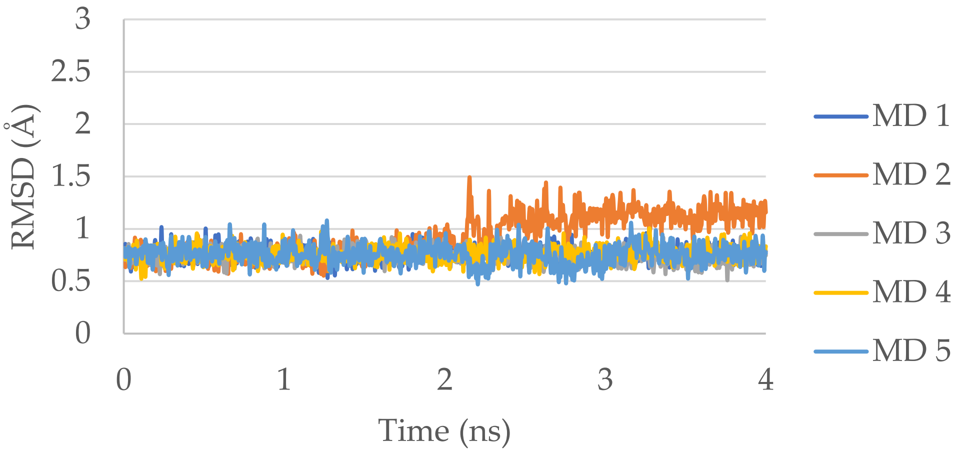

Figure S10. Ligand RMSD vs. time for the GSTP1-compound **9** complex (docking pose 2).

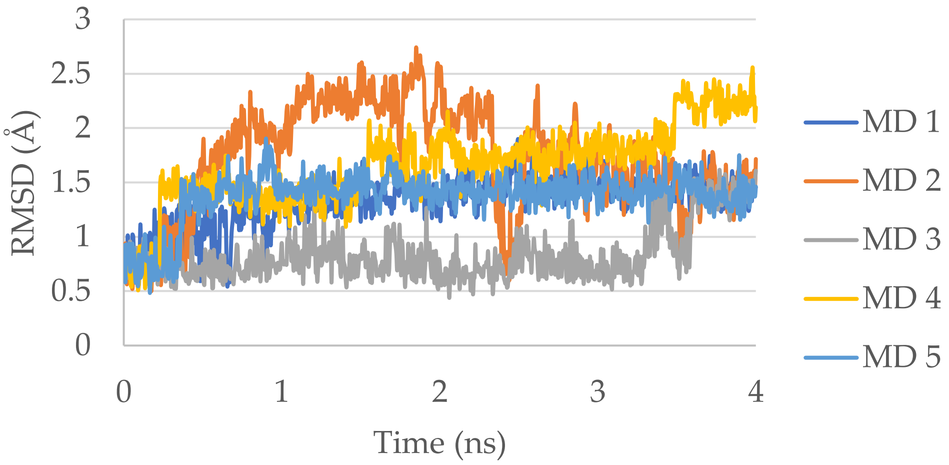

Figure S11. Ligand RMSD vs. time for the MCL1-compound **9** complex (docking pose 1).

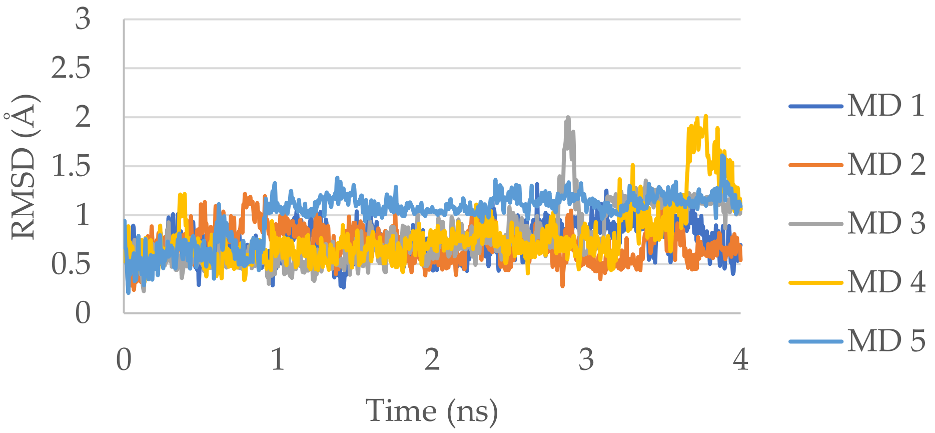

Figure S12. Ligand RMSD vs. time for the MCL1-compound **9** complex (docking pose 2).

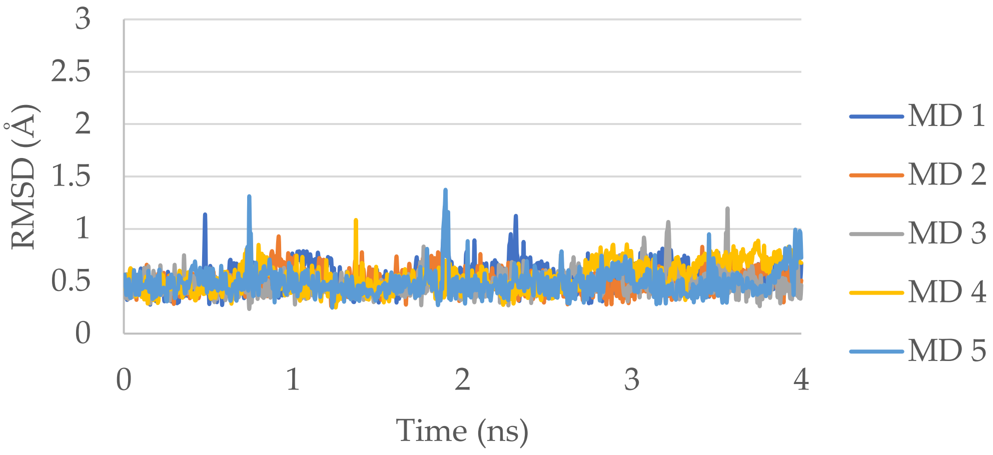

Figure S13. Ligand RMSD vs. time for the MCL1-compound **9** complex (docking pose 3).

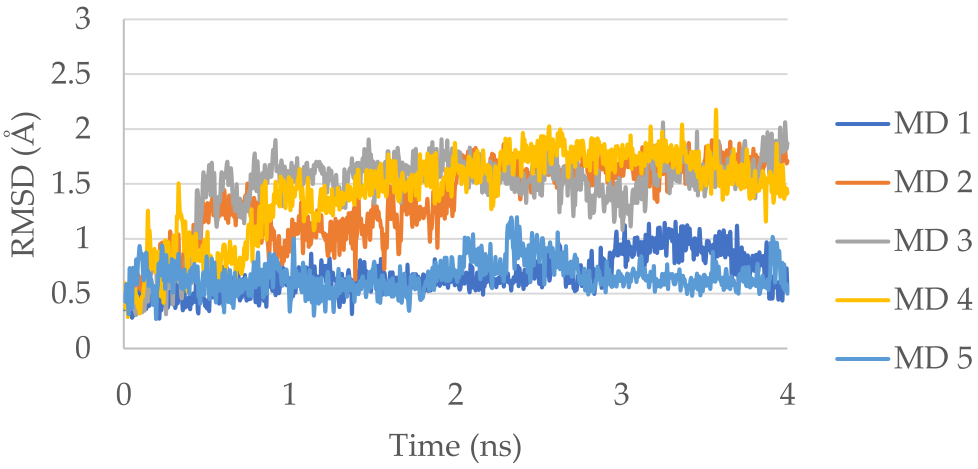

Figure S14. Ligand RMSD vs. time for the MMP2-compound **9** complex (docking pose 1).

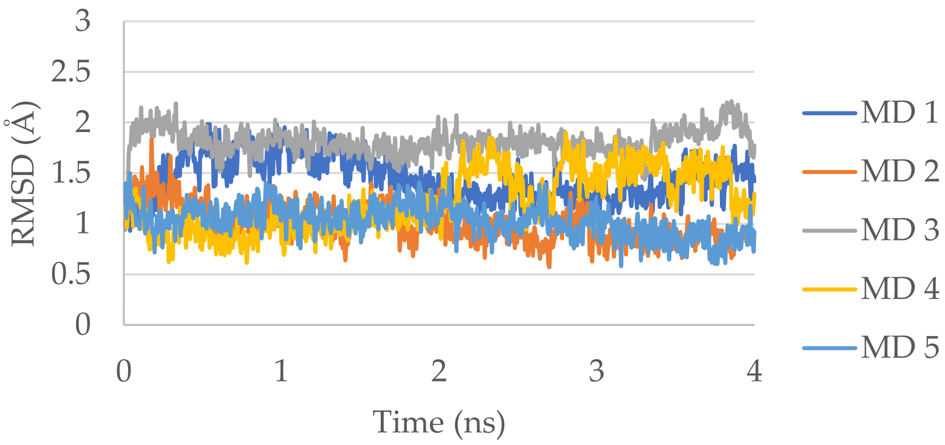

Figure S15. Ligand RMSD vs. time for the MMP2-compound **9** complex (docking pose 2).

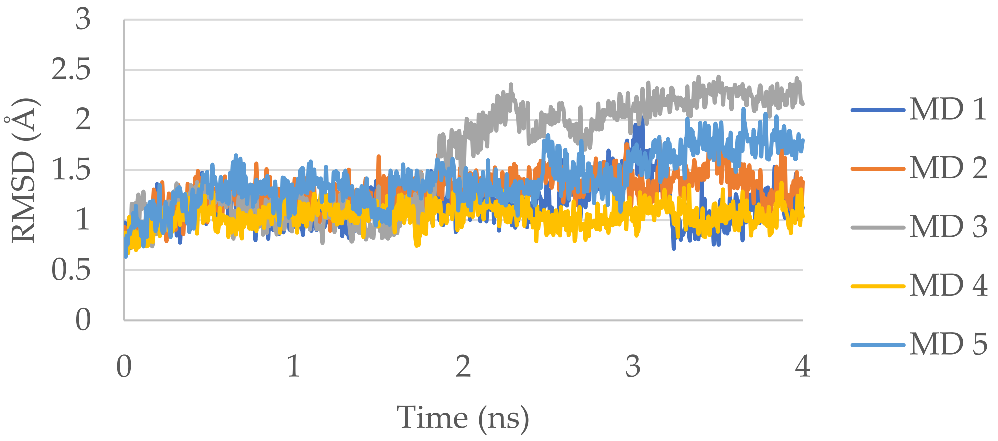

Figure S16. Ligand RMSD vs. time for the MMP2-compound **9** complex (docking pose 3).

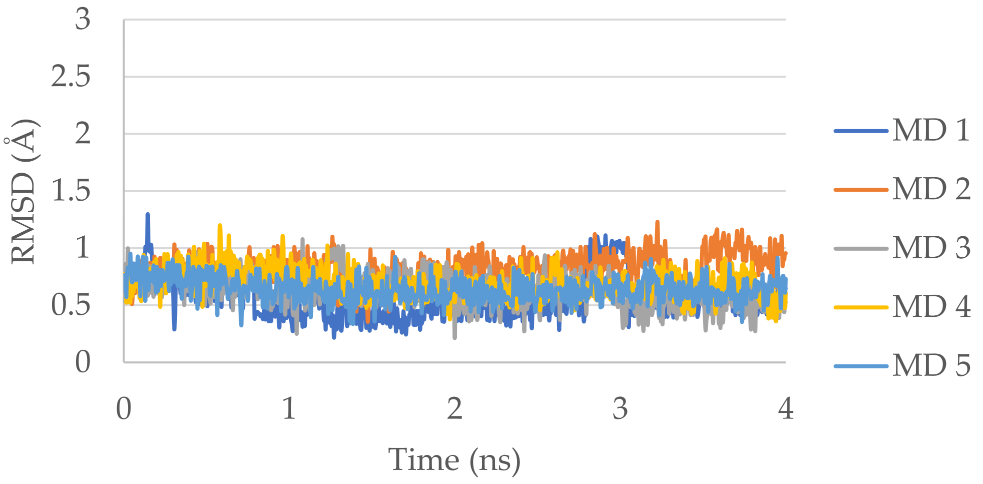

Figure S17. Ligand RMSD vs. time for the MTNR1B-compound **9** complex (docking pose 1).

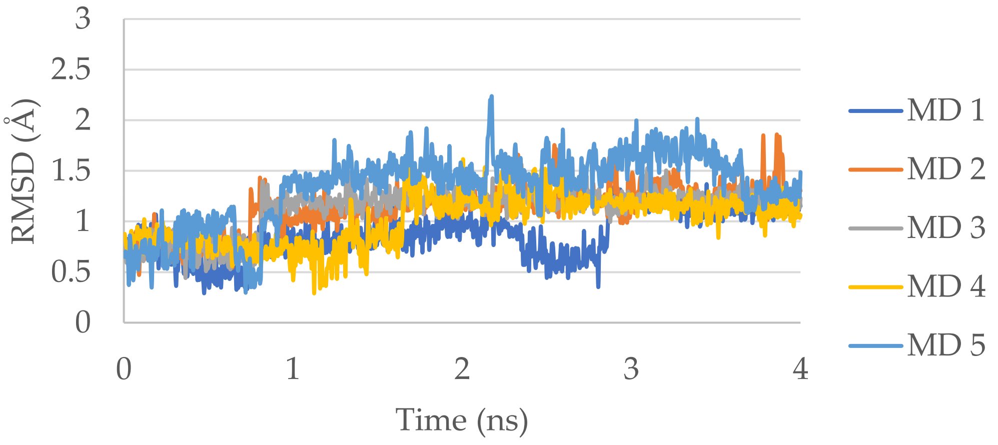

Figure S18. Ligand RMSD vs. time for the MTNR1B-compound **9** complex (docking pose 2).

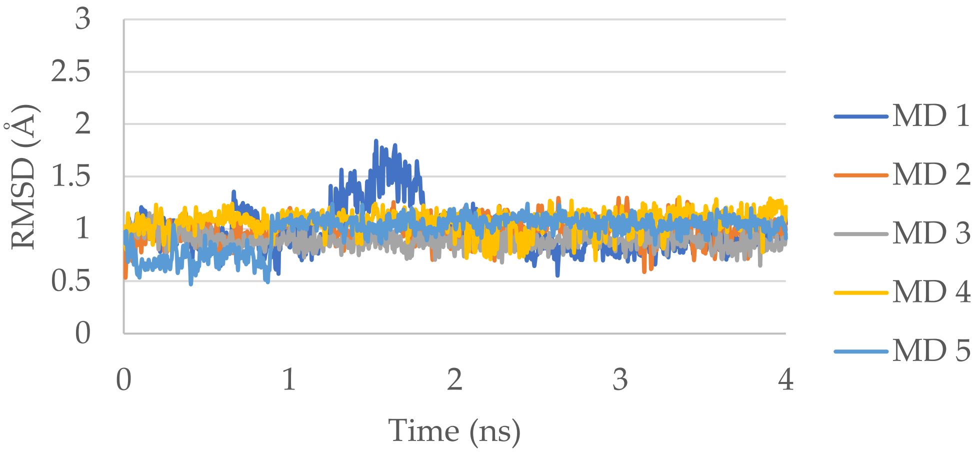

Figure S19. Ligand RMSD vs. time for the mTOR-compound **9** complex (docking pose 1).

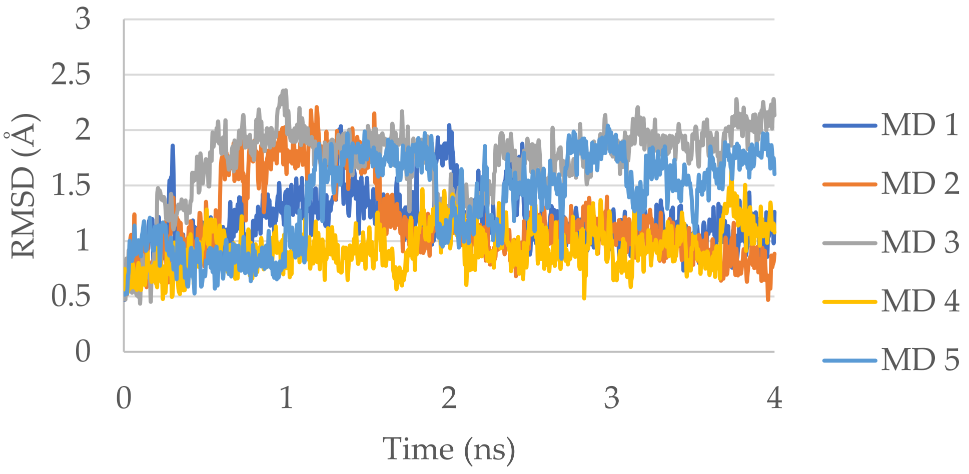

Figure S20. Ligand RMSD vs. time for the NFKB-compound **9** complex (docking pose 1).

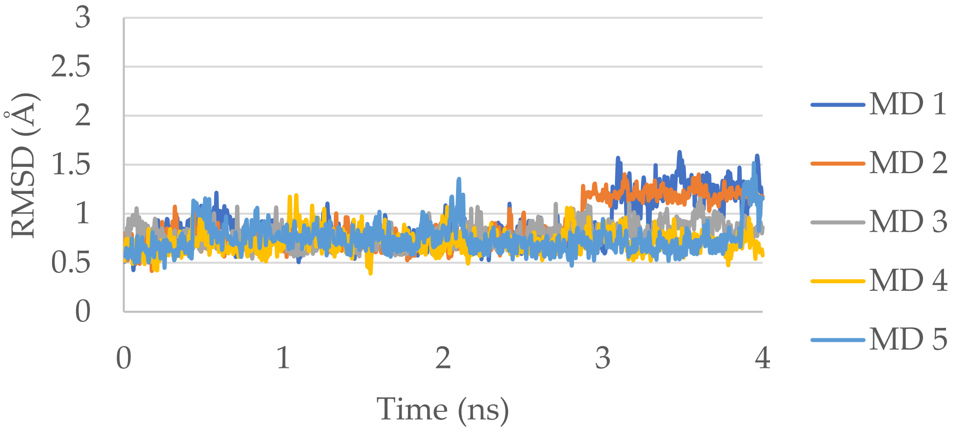

Figure S21. Ligand RMSD vs. time for the NFKB-compound **9** complex (docking pose 2).

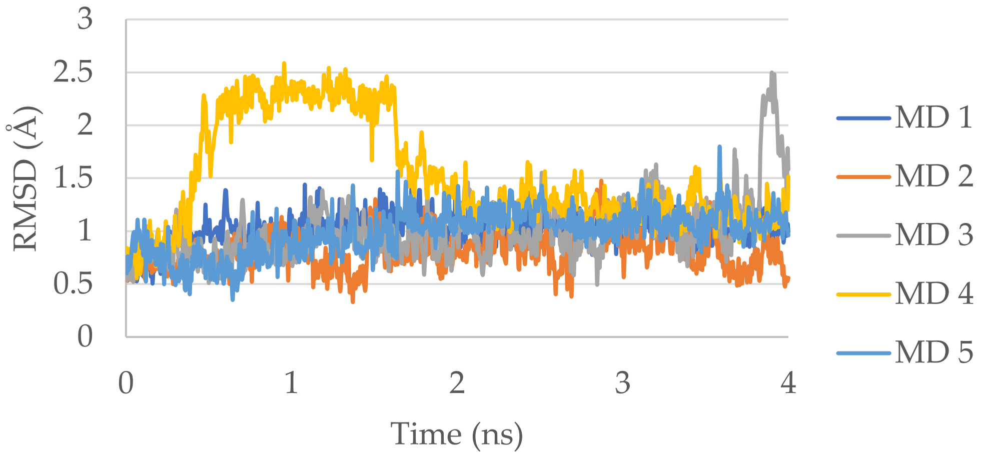

Figure S22. Ligand RMSD vs. time for the NFKB-compound **9** complex (docking pose 3).

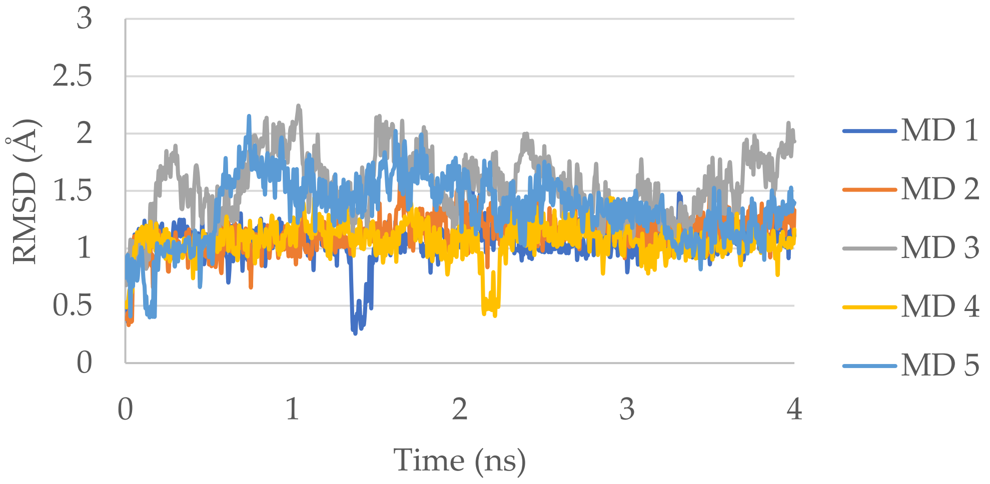

Figure S23. Ligand RMSD vs. time for the PI3KA-compound **9** complex (docking pose 1).

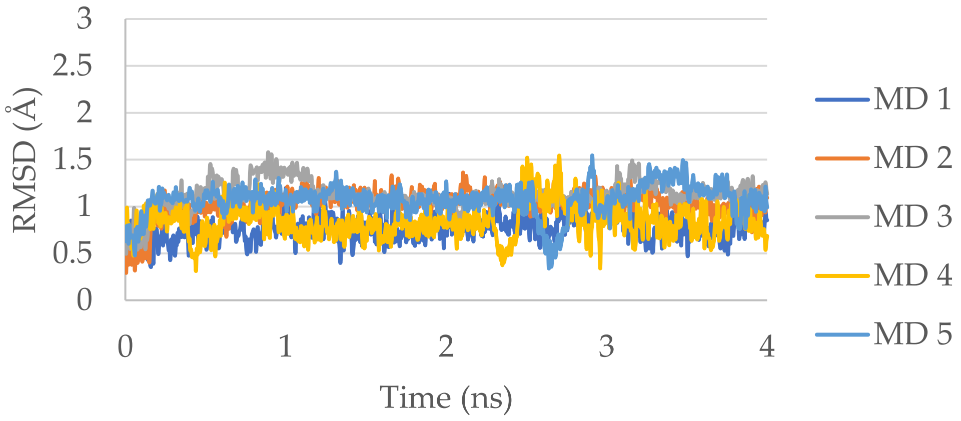

Figure S24. Ligand RMSD vs. time for the PI3KA-compound **9** complex (docking pose 2).

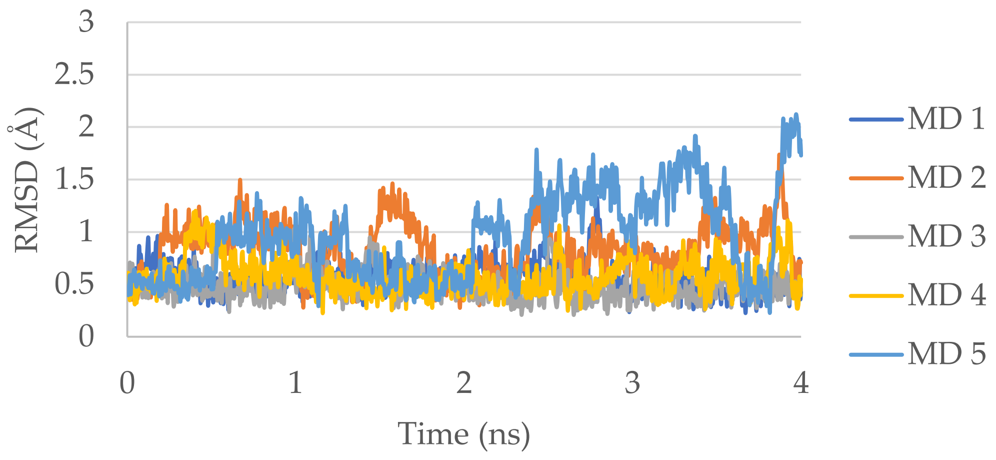

Figure S25. Ligand RMSD vs. time for the PI3KA-compound **9** complex (docking pose 3).

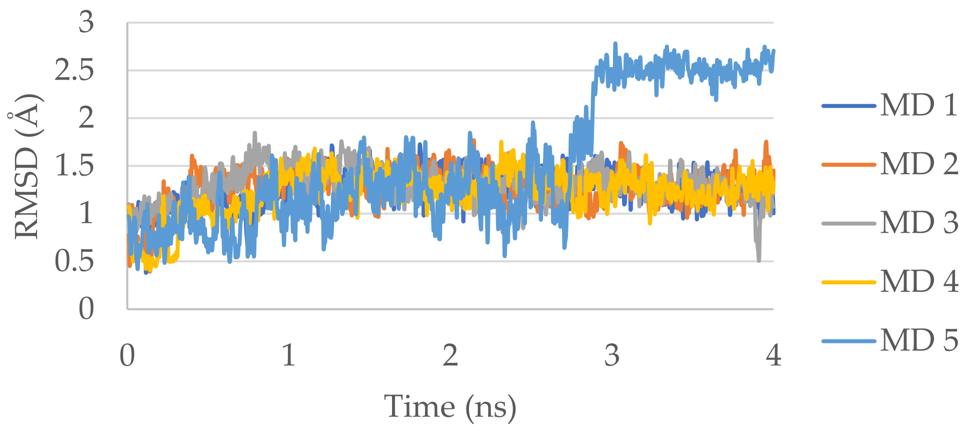

Figure S26. Ligand RMSD vs. time for the PI3KA-compound **9** complex (docking pose 4).

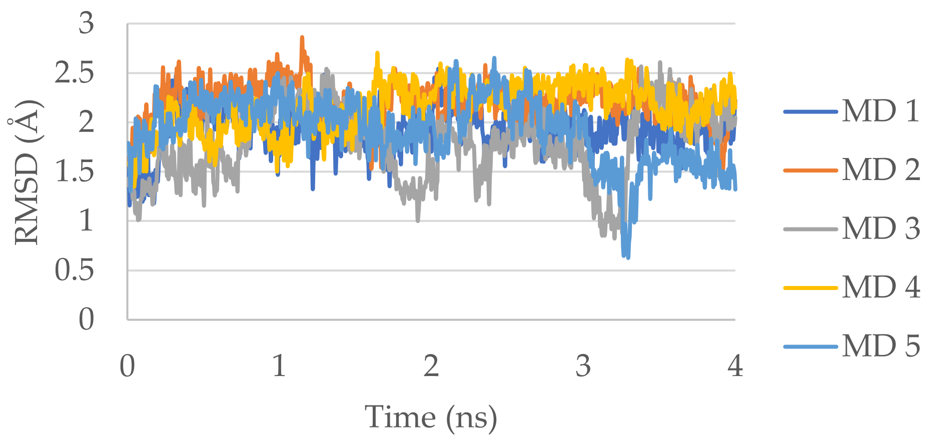

Figure S27. Ligand RMSD vs. time for the RELA-compound **9** complex (docking pose 1).

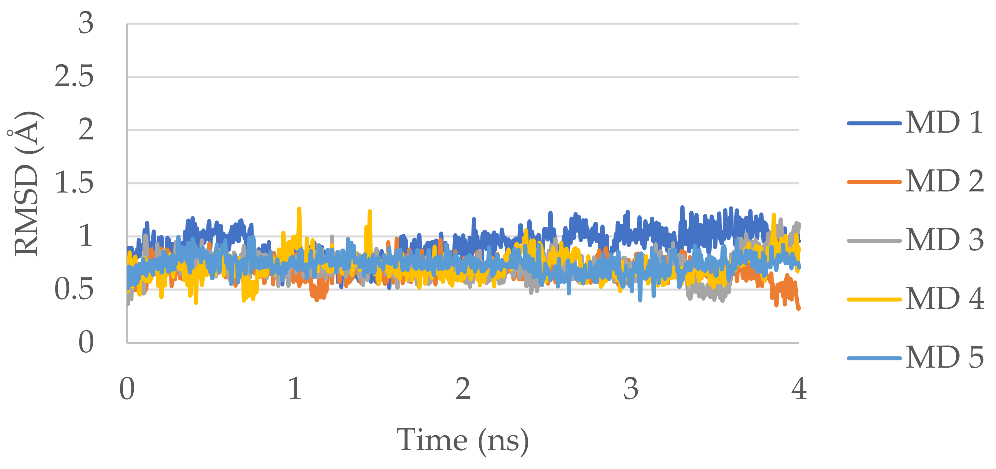

Figure S28. Ligand RMSD vs. time for the RIPK2 -compound **9** complex (docking pose 1).

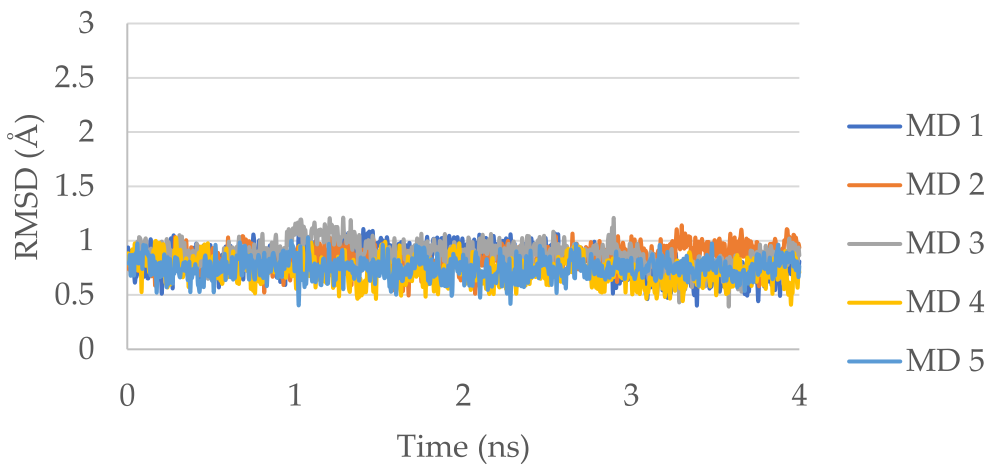

Figure S29. Ligand RMSD vs. time for the RIPK2 -compound **9** complex (docking pose 2).

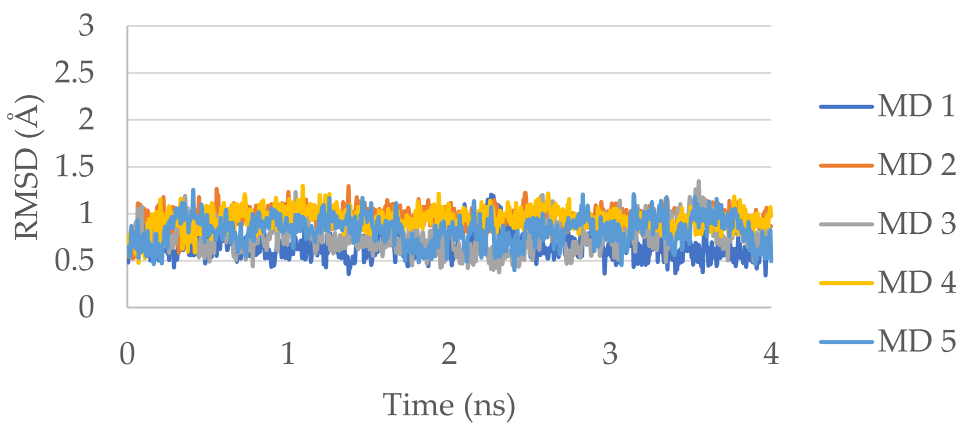

Figure S30. Ligand RMSD vs. time for the RIPK2 -compound **9** complex (docking pose 3).

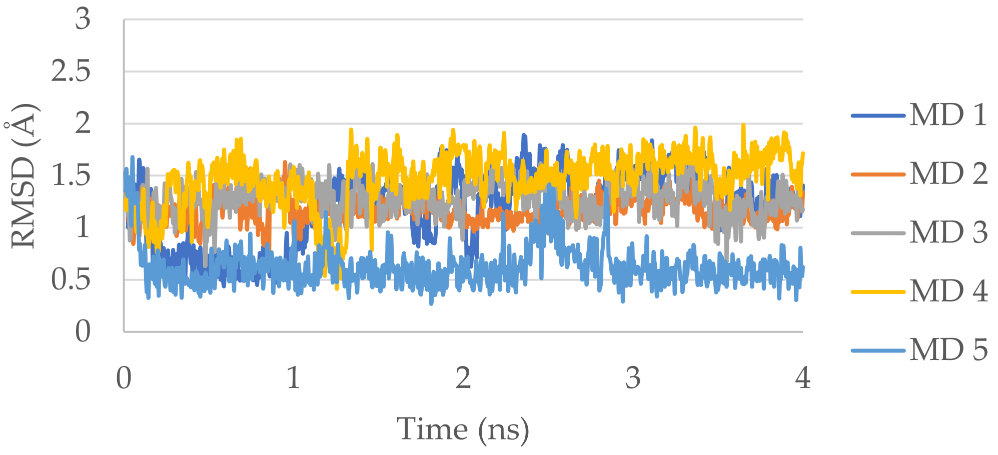

Figure S31. Ligand RMSD vs. time for the STAT3 -compound **9** complex (docking pose 1).

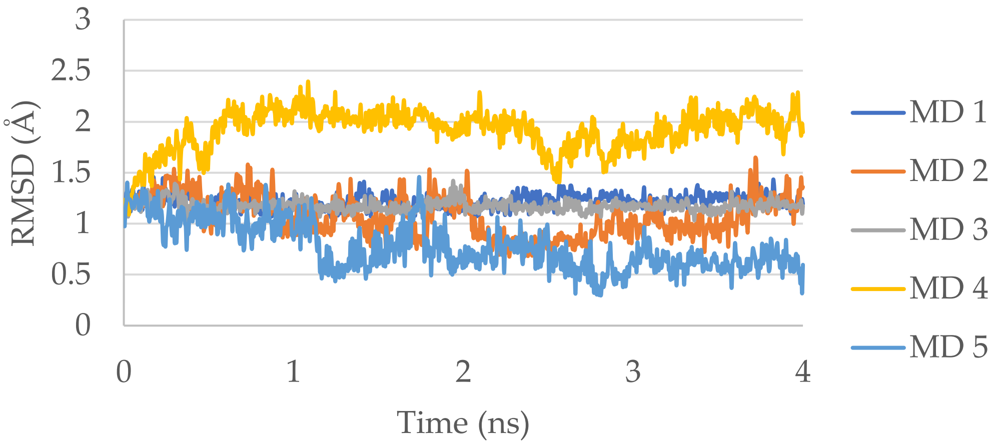

Figure S32. Ligand RMSD vs. time for the STAT3 -compound **9** complex (docking pose 2).

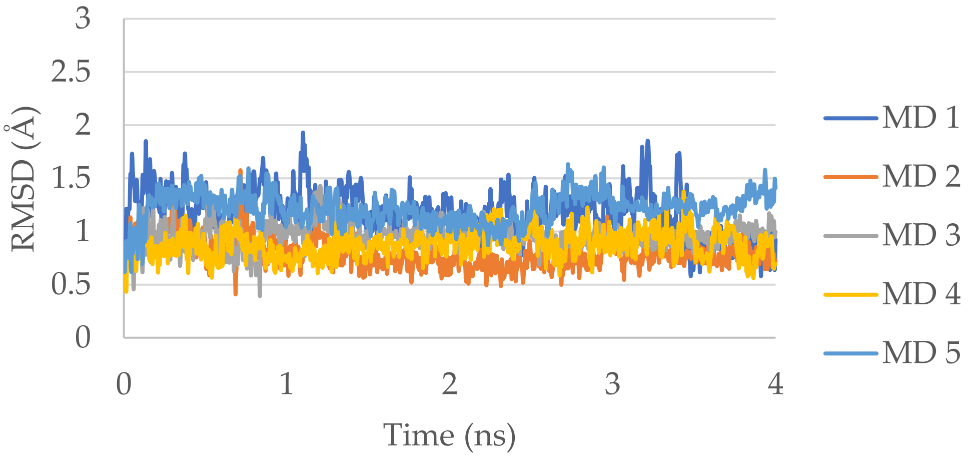

Figure S33. Ligand RMSD vs. time for the STAT3 -compound **9** complex (docking pose 3).
